# ING5 Is Phosphorylated by CDK2 and Controls Cell Proliferation Independently of p53

**DOI:** 10.1371/journal.pone.0123736

**Published:** 2015-04-10

**Authors:** Ulrike Linzen, Richard Lilischkis, Ruwin Pandithage, Britta Schilling, Andrea Ullius, Juliane Lüscher-Firzlaff, Elisabeth Kremmer, Bernhard Lüscher, Jörg Vervoorts

**Affiliations:** 1 Institute of Biochemistry and Molecular Biology, Medical School, RWTH Aachen University, Pauwelsstrasse 30, 52057, Aachen, Germany; 2 Helmholtz Zentrum München, Institute of Molecular Immunology, Marchioninistrasse 25, 81377, München, Germany; Università di Palermo, ITALY

## Abstract

Inhibitor of growth (ING) proteins have multiple functions in the control of cell proliferation, mainly by regulating processes associated with chromatin regulation and gene expression. ING5 has been described to regulate aspects of gene transcription and replication. Moreover deregulation of ING5 is observed in different tumors, potentially functioning as a tumor suppressor. Gene transcription in late G1 and in S phase and replication is regulated by cyclin-dependent kinase 2 (CDK2) in complex with cyclin E or cyclin A. CDK2 complexes phosphorylate and regulate several substrate proteins relevant for overcoming the restriction point and promoting S phase. We have identified ING5 as a novel CDK2 substrate. ING5 is phosphorylated at a single site, threonine 152, by cyclin E/CDK2 and cyclin A/CDK2 *in vitro*. This site is also phosphorylated in cells in a cell cycle dependent manner, consistent with it being a CDK2 substrate. Furthermore overexpression of cyclin E/CDK2 stimulates while the CDK2 inhibitor p27^KIP1^ represses phosphorylation at threonine 152. This site is located in a bipartite nuclear localization sequence but its phosphorylation was not sufficient to deregulate the subcellular localization of ING5. Although ING5 interacts with the tumor suppressor p53, we could not establish p53-dependent regulation of cell proliferation by ING5 and by phospho-site mutants. Instead we observed that the knockdown of ING5 resulted in a strong reduction of proliferation in different tumor cell lines, irrespective of the p53 status. This inhibition of proliferation was at least in part due to the induction of apoptosis. In summary we identified a phosphorylation site at threonine 152 of ING5 that is cell cycle regulated and we observed that ING5 is necessary for tumor cell proliferation, without any apparent dependency on the tumor suppressor p53.

## Introduction

The cell cycle of eukaryotic cells is controlled by cyclin-dependent kinases (CDKs) [1]. These enzymes integrate signals that emanate from intracellular and extracellular cues and regulate cell cycle progression and cell proliferation. Deregulation of the cell cycle can lead to increased cellular proliferation, a hallmark of the development and progression of human cancer [2]. The activity of CDKs is controlled by the cell cycle-dependent accumulation and subsequent proteolytic degradation of the cyclin subunits, positive and negative phosphorylation of the CDK subunits as well as through interactions with CDK inhibitors [3,4]. The inappropriate activation of CDKs contributes to tumorigenesis [5,6].

Cyclin E/CDK2 controls the G1-S phase transition by phosphorylating a number of substrates associated with gene transcription, including the retinoblastoma protein RB [7], p53 [8], FoxM1 [9], NPAT [10], E2F-5 [11] among others [12]. In particular the phosphorylation of RB and NPAT favors the expression of genes, such as cyclin E and histone genes, which are important for initiation of S phase and feedback control [13,14,15]. Cyclin E/CDK2 also regulates the stability of p27^KIP1^, which interacts with the kinase complex and negatively controls its activity [16,17]. Moreover cyclin E/CDK2 affects replication and duplication of centrosomes [18,19]. Cyclin A/CDK2 has overlapping functions with cyclin E/CDK2, including functions during replication [20,21,22,23]. Cyclin A/CDK2 acts also later in the cell cycle, e.g. by contributing to progression into mitosis [24,25,26,27], and is involved in DNA repair processes [28,29,30]. A number of cyclin E/CDK2 substrates have been identified, with some also phosphorylated by cyclin A/CDK2 [31,32]. Together these findings document a key role of CDK2 complexes in regulating the progression through the cell cycle and in controlling replication.

We have reported on a cyclin E/CDK2 screen that identified 26 proteins as potential substrates [32]. We studied the NAD^+^-dependent deacetylase SIRT2 and determined that the enzymatic activity of this protein is negatively regulated by cyclin E/CDK2-dependent phosphorylation both in vitro and in cells. The phosphorylation site is located in an unstructured C-terminal extension of the catalytic domain of SIRT2. Other sirtuins have also potential CDK phosphorylation sites at similar positions relative to the catalytic domain [33]. An additional potential substrate identified in the screen was the inhibitor of growth protein 5 (ING5) [32]. ING1, the founding member of the ING family, was found in a screen for differentially expressed mRNAs between control tissue and breast cancer cell lines and, as the name implies, inhibits cell proliferation [34]. Subsequently ING2-5 were described, all of which have been found deregulated in at least some cancers [35,36,37]. Moreover ING proteins affect aging, apoptosis and DNA repair [37,38,39,40].

ING5 was identified in a homology search for additional ING family members [41]. Similar to other ING proteins, ING5 possesses different structural and functional elements, including a PHD (plant homeodomain), an NCR (novel conserved region), a LZL (leucine zipper like) finger, and two NLS (nuclear localization sequence) motifs. ING proteins bind to methylated core histones through their PHD motif, notably ING5 reads histone H3 when di- or trimethylated at lysine 4 (H3K4me2/3) [42,43,44]. ING5 is a subunit of two distinct histone acetyltransferase (HAT) complexes with HBO1 or MOZ/MORF as the two catalytic subunits [45]. These complexes contain two different alternative scaffolding proteins, i.e. a BRPF protein or a JADE protein, that determine their specificity towards different core histones [46,47,48]. Together with the observation that ING5 associates with MCM2 [45], these findings suggest a role of ING5 in replication [49,50]. In addition these complexes appear to participate in transcription, for example by interacting with transcriptional regulators such as the tumor suppressor p53 and through the ability of ING5 to recognize H3K4me3, a mark associated with open chromatin and transcribed genes [51].

Several studies have implicated ING5 in cancer development. For example the region on chromosome 2q37.3 where *ING5* is located is deleted in oral squamous cell carcinoma and odontogenic tumors [52,53,54]. In head and neck squamous cell carcinoma the subcellular localization of ING5 was reported to be cytosolic in some tumors [55]. Moreover cytosolic ING5 is positively correlated with progression of gastric cancer [56]. It suggests that this is a potential mechanism to interfere with ING5 function as cytosolic ING5 is less likely to impact replication and transcription. In pancreatic tumors miR-196a levels correlate inversely with survival of patients. One of the targets of this miRNA is ING5, suggesting that repression of ING5 correlates with poor survival. miRNA-196a reduces apoptosis, enhances invasion and proliferation of tumor cells. However whether these effects are dependent on ING5 deregulation is not entirely clear [57]. In another study the silencing of ING5 caused sensitivity to tamoxifen in MCF7 breast cancer cells, indicating that repression of ING5 expression is associated with tumor cell progression [58]. These findings imply a tumor suppressor function of ING5, but most of these studies are of correlative nature.

Similar to other ING proteins, ING5 has been reported to interact with p53 [41]. By enhancing acetylation of p53 in a p300-dependent manner, ING5 stimulates p53-dependent gene transcription. Moreover ING5 functions as a TIP60 co-factor that promotes p53 acetylation and transcriptional activity in response to DNA damage [59]. The anti-proliferative effect of ING5 depends also on INCA1 [60]. INCA1 was identified previously as an interaction partner of cyclin A/CDK2 [61]. In epidermal stem cells indirect evidence suggests that ING5 interacts with p63, a p53 related factor, that is involved in the self-renewal of epidermal stem cells [62,63]. The knockdown of ING5 promotes differentiation, suggesting that in these non-tumorigenic cells ING5 is important to maintain a self-renewing, non-differentiated state. Similar to the findings described above, ING5 is associated with components of a MORF complex in epidermal stem cells [63]. This indicates that in these primary cells ING5 is associated with sustained proliferation, which differs from the apparent role of ING5 in tumor cells. This is also compatible with ING5’s role as an important factor in DNA replication.

Here we describe ING5 as a novel cyclin E/CDK2 substrate. ING5 is phosphorylated at a single site, threonine 152 (T152). We have addressed the functional relevance of T152 phosphorylation by generating non-phosphorylatable and phospho-mimicking mutants and determined their effect on cell proliferation dependent on the tumor suppressor p53. We find that ING5 is required for cell proliferation independently of p53 and that phosphorylation of T152 does not impinge on cell proliferation.

## Materials and Methods

### Cells, transfections and assays

HEK293, U2OS, T98G and HeLa cells were cultured in DMEM supplemented with penicillin/streptomycin and 10% fetal calf serum. For MCF7 cells this medium was supplemented with pyruvate and glutamine. HCT116 cells were cultured in McCoy medium supplemented with penicillin/streptomycin and 10% fetal calf serum. Transient transfection assays were performed as described previously [9]. For cell cycle analysis T98G cells were seeded at a density of 1.2 x 10^6^ cells/10 cm dish and serum starved in 0.1% FCS the next day. After 72h the cells were split 1:3 and fed with medium containing 10% FCS, which promotes reentry into the cell cycle [64]. For colony formation assays, cells were plated at a density of 2.5 x 10^5^ cells/6cm plate. Cells were co-transfected, using ExGen500 (MBI Fermentas), with 4.5 μg expression plasmids encoding p27^KIP1^, ING5, and ING5 mutants or the pEVRF0-HA control vector and 0.5 μg pBABEpuro, a puromycin resistance plasmid. Cells transfected with 5 μg pEVRF0-HA only were used as a negative control. Media was changed six hours after transfection and three hours later cells were selected for sixteen hours with 2 μg/ml puromycin. Ten to twelve days after transfection, plates were washed with PBS and stained in 0.2% (w/v) methylene blue in methanol. Images of each plate were converted to grayscale using Adobe Photoshop 7.0. The numbers of gray pixels were determined and the values of the negative controls were subtracted to determine colony density. For the cyclin E and cyclin A knockdown experiments, ING5 expressing plasmids were transfected first using the Calcium Phosphate method. The next day the cells were split 1:3 and the siRNAs (final concentration 20 nM) were reverse transfected using 20 μl HiPerfect/10 cm dish (Qiagen). The following Dharmacon human siGenome SMARTpools were used: cyclin E (M-003213-02), cyclin A (M-003205-02) and control (D-001206-14). Roscovitine and colcemid were purchased from Calbiochem, fulvestrant, tamoxifen and nocodazole from Sigma.

### Plasmid constructs

All ING5 constructs were generated by recombining the PCR-amplified ORF into pDonR201 of Invitrogen's Gateway cloning system by virtue of attB1 and attB2-mediated BP-clonase reaction. A subsequent LR-recombination reaction with Gateway-compatible pGex4T2, and pEVRF0-HA resulted in GST- and HA-tagged expression constructs. Site-directed mutagenesis was carried out using the Quick Change protocol (Stratagene) to generate the T152A and T152D mutants. All constructs were verified by DNA sequencing. pCMV-EGFP-tubulin was cloned by standard procedures and used to control transfection efficiency and to identify the transfected cells. For constructs expressing siRNA, the following sequences were cloned into pSUPER [65]:

siING5-1: 5’-GGA AGA TAA GAA AGC AGA GAT AGA CAT ACT AGC TGC AGA GTA CAT CTC CA (sense) and 5’-TGG AGA TGT ACT CTG CAG CTA GTA TGT CTA TCT CTG CTT TCT TAT CTT CC (antisense);

siING5-2: 5’-GAT CCA GAA CGC CTA CAG CAA ATG TAA AGA ATA CAG TGA CGA CAA AGT G (sense) and 5’-CAC TTT GTC GTC ACT GTA TTC TTT ACA TTT GCT GTA GGC GTT CTG GAT C (antisense);

Additional constructs for cyclins and CDKs have been described elsewhere [32].

### Preparation of active cyclin/CDK complexes, *in vitro* kinase assays, *in vivo* labeling, and phosphatase assays

Expression of human cyclin/CDK complexes occurred in insect cells upon infection with recombinant baculo viruses. The complexes were purified using the GST tag of the cyclins. These procedures and in vitro kinase assays have been described [32,66]. For in vivo labeling, HEK293 cells were transiently transfected and two days later metabolically labeled in phosphate-free DMEM supplemented with 10% dialyzed fetal calf serum, 20 mM sodium bicarbonate, 18 mM Hepes pH 7.5 and 10–20 MBq of [32P]-orthophosphate for 2 hours. The labeled cells were lysed in RIPA buffer (10 mM Tris-HCl pH 7.4, 150 mM NaCl, 1% NP-40, 1% desoxycholate, 0.1% SDS, 7 μg/ml aprotinin and 20 mM β-glycerophosphate). The HA-tagged ING5 proteins were immunoprecipitated with α-HA mAb (3F10) and protein G-agarose. The precipitates were washed four times in the same buffer and separated by SDS-PAGE on 12% gels. For efficient inhibition of phosphatases we supplemented all buffers with 3 mM Na_3_VO_4_, 30 mM NaF, and 30 mM β-glycerophosphate.

For phosphatase treatment cells were lysed in RIPA buffer and ING5 immunoprecipitated. Immobilized proteins were resuspended in 50 mM Tris-HCl, pH 8.5, and 1 mM MgCl_2_. The probes were then incubated in the presence of 2.5 U of shrimps alkaline phosphatase (Sigma) at 30°C for 30 min. The reactions were stopped by adding SDS-sample buffer.

### Antibodies, immunoprecipitation and Western blotting

The rabbit polyclonal anti-ING5 antiserum and the rat monoclonal antibodies 7A11 were raised against a GST-ING5 fusion protein and the rat monoclonal antibodies 3H5 against a peptide phosphorylated at T152.

The ING5 pAb 3716 (Abcam), CDK2 pAb H-298 (Santa Cruz), cyclin E pAb M-20 and mAb H-12 (Santa Cruz), cyclin A mAb BF638 (Santa Cruz), cyclin B H20 (Santa Cruz), RB/RB-P Kit #9969 (Cell Signaling), the actin mAb C4 (MP Biomedicals), ASH2L mAb 4C5 (Activmotif) [67], and the HA-tag mAB 3F10 (Roche) are commercially available. Low stringency (co)-immunoprecipitations, GST pull-down assays, and Western blotting were done as described previously [68]. For low stringency lysates cells were lysed in F-buffer (10 mM Tris-HCl, pH 7.05, 50 mM NaCl, 30 mM Na_4_P_2_O_7_, 50 mM NaF, 5 μM ZnCl_2_, 100 μM Na_3_VO_4_, 1% Triton X-100, 1 mM phenylmethylsulfonyl fluoride, 5 units/ml α2-macroglobulin, 2.5 units/ml pepstatin A, 2.5 units/ml leupeptin, 0.15 mM benzamidine), for high stringency lysates in RIPA buffer (as described above).

### FACS

For life cell FACS analysis, 1x10^6^/ml cells were harvested and stained with Vybrant DyeCycle violet stain (Molecular Probes). Apoptotic cells were identified by staining with Annexin V-eFluor 450 (eBioscience). Five million cells/ml were diluted in 1 ml binding buffer (eBioscience) and 100 μl of this suspension was incubated with 5 μl Annexin V for 15 min. Subsequently dead cells were stained by diluting the cell suspension with 200 μl binding buffer (eBioscience) and 5 μl PI (propidium iodide, eBioscience). Cells were analyzed on a facscanto II and evaluated using the FlowJo software.

### Immunocytochemistry

HeLa cells transiently transfected with constructs expressing HA-tagged ING5 proteins were fixed in PBS containing 3.7% paraformaldehyde for 15 min followed by washing and permeabilization in PBS with 0.1% Triton-X-100 for 5 min. The samples were blocked in PBS with 2% BSA and incubated with the mAb 3F10 as primary antibody. The secondary antibody was conjugated to Alexa555 (Invitrogen). The DNA was stained with Hoechst 33258.

## Results

### ING5 is a novel cyclin E/CDK2 substrate

**Fig 1:**
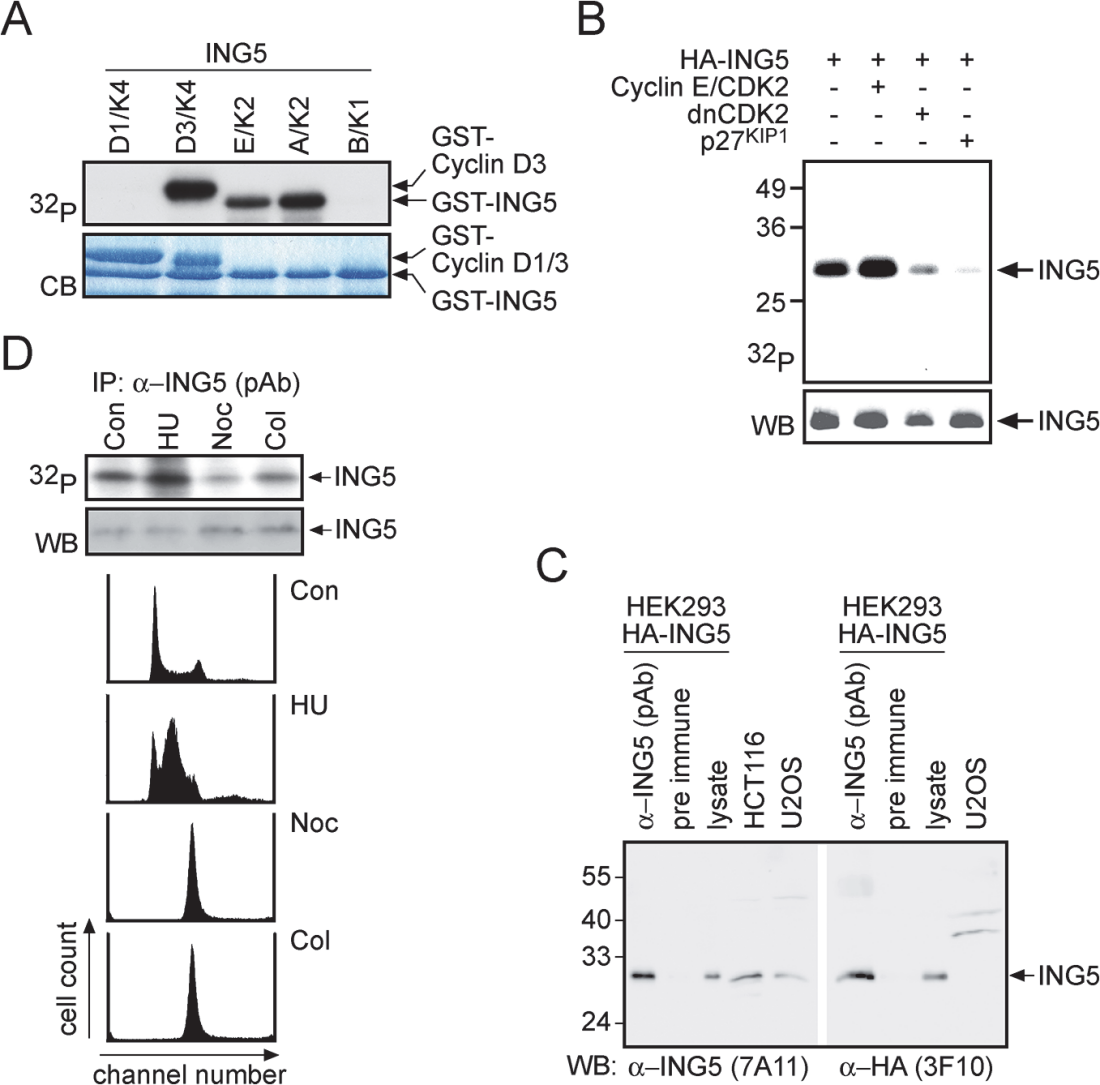
ING5 is a CDK2 substrate. (A) Bacterially expressed and purified GST-tagged ING5 was incubated with 12.5 fkatal of the indicated purified cyclin/CDK complexes in the presence of ^32^P-γ-ATP. The proteins were resolved by 7–17% SDS-PAGE and visualized by autoradiography (^32^P, upper panel) or Coomassie blue staining (CB, lower panel). (B) HEK293 cells were transiently transfected with plasmids encoding HA-ING5 (10 μg), cyclin E/CDK2 (500 ng each), a catalytically inactive form of CDK2 (dnCDK2, 5 μg) and p27^KIP1^ (5 μg) as indicated. The cells were metabolically labeled with ^32^P-orthophosphate and ING5 was then immunoprecipitated by virtue of its HA-tag. The top panel shows an autoradiograph, the bottom panel a Western blot using mAb 3F10 (HA-tag). (C) HA-ING5 was expressed in HEK293 cells and immunoprecipitated using the ING5-specific polyclonal serum (pAb #3716) or a pre-immune serum. In addition aliquots of total cell lysates of transiently transfected HEK293 cells or of two tumor cell lines (HCT116 and U2OS) were analyzed as indicated. Parallel Western blots were developed using mAb 7A11 (ING5) and mAb 3F10 (HA-tag). (D) HEK293 cells were left untreated or treated with 200 μM hydroxyurea (HU), 400 ng/ml nocodazole (Noc) or 200 ng/ml colcemid (Col) for 18 hrs and then metabolically labeled with ^32^P-orthophosphate. Endogenous ING5 protein was immunoprecipitated from RIPA buffer lysates using the ING5-specific polyclonal serum (#3716). The top panel shows an autoradiograph, the bottom panel a Western blot using mAb 7A11 (ING5). The FACS profiles of the cells treated with the different cell cycle inhibitors are shown below.

In a screen to identify novel cyclin E/CDK2 substrates, we used high-density protein arrays that were phosphorylated with insect cell expressed and purified cyclin E/CDK2 in the presence of ^32^P-γ-ATP [32]. One protein identified in this screen was ING5. The finding was verified using recombinant GST-ING5, which was phosphorylated by cyclin E/CDK2 and cyclin A/CDK2 but not by G1 and M phase cyclin complexes (Fig 1A). The activities of all CDK complexes were verified [32]. To evaluate whether ING5 is phosphorylated in cells, an HA-tagged version of ING5 was expressed transiently in HEK293 cells that were subsequently metabolically labeled with ^32^P-orthophosphate. Immunoprecipitation of ING5 revealed that this protein was phosphorylated in cells (Fig 1B). Co-expression of cyclin E/CDK2 enhanced while the expression of a dominant negative form of CDK2 (dnCDK2) or of the CDK2 inhibitor p27^KIP1^ repressed ING5 phosphorylation (Fig 1B). These findings are compatible with the hypothesis that ING5 is a cyclin E/CDK2 substrate in cells.

To study ING5 we generated both mono- and polyclonal antibodies. The polyclonal serum (pAb) immunoprecipitated overexpressed as well as endogenous ING5 and the mAb 7A11 recognized these proteins on immunoblots (Fig 1C). Using these reagents we evaluated endogenous ING5 phosphorylation. HCT116 cells arrested in early S phase with hydroxyurea (HU) or in mitosis with nocodazole (Noc) or colcemid (Col), as determined by FACS analysis (Fig 1D), were labeled with ^32^P-orthophosphate. In comparison to the untreated control, overall phosphorylation of ING5 increased in the HU-treated cells and decreased in mitotic cells (Fig 1D, upper panel). This suggested that ING5 is phosphorylated preferentially in late G1, S and G2 phase, in agreement with being modified by CDK2.

**Fig 2:**
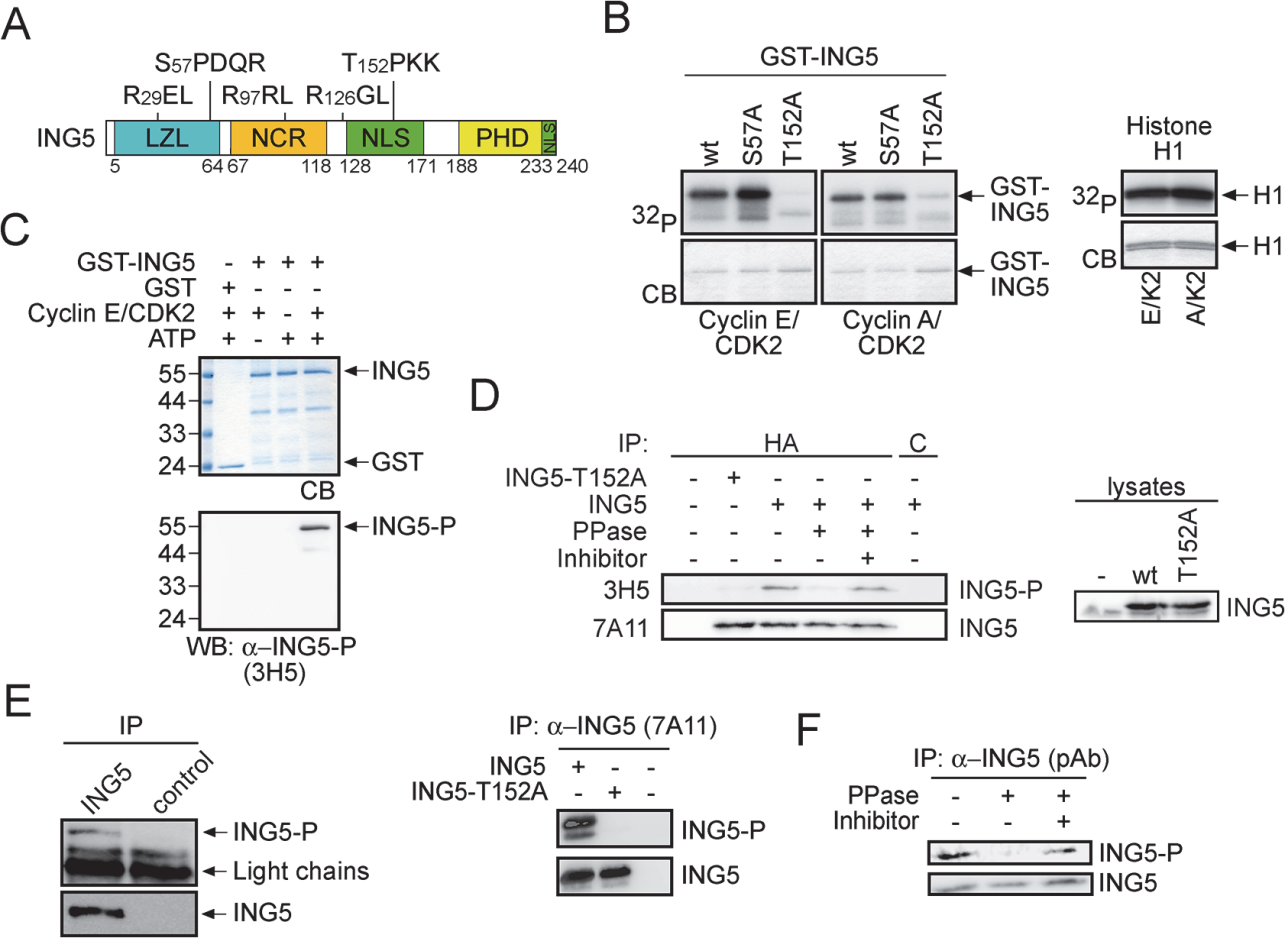
ING5 is phosphorylated at Thr152 by cyclin E/CDK2. (A) Schematic drawing of the ING5 protein. The numbers refer to amino acid positions. LZL, leucine-zipper-like region (blue); NCR, novel conserved region (orange); NLS, nuclear localization signals (green, a bipartite NLS from amino acid 128–171 and a basic NLS at the C-terminus); PHD, C-terminal plant homeodomain zinc finger (yellow); the two potential CDK phosphorylation sites at S57 and at T152, and three RXL motifs, potential cyclin binding sites, are indicated [35,36,37].(B) Recombinant GST-ING5, GST-ING5-S57A, and GST-ING5-T152A were incubated with baculovirus-derived cyclin E/CDK2 and cyclin A/CDK2 in the presence of ^32^P-γ-ATP. For control kinase assays were performed with histone H1. CB, Coomassie blue stained gels; ^32^P, autoradiographies.(C) GST or GST-ING5 were incubated with cyclin E/CDK2 in the presence or absence of ATP. Western blot analysis was performed using mAb 3H5, which was generated against a phospho-peptide containing phosphorylated T152. CB, Coomassie blue stained gel.(D) ING5 or ING5-T152A were expressed transiently in HEK293 cells. ING5 proteins were immunoprecipitated using a rat monoclonal HA-antibody (3F10) or for control a rat monoclonal ASH2L antibody (4C5), treated with our without shrimps alkaline phosphatase in the presence or absence of phosphatase inhibitor as indicated. Phosphorylation at T152 was measured on Western blots using mAb 3H5. Total levels of ING5 were determined with mAb 7A11.(E) Endogens ING5 from HEK 293 cells was immunoprecipitated using an ING5 antibody (7A11) or for control an ASH2L antibody (4C5). Phosphorylation at T152 was measured on Western blots using mAb 3H5. Total levels of ING5 were determined with mAb 7A11 (left panel). ING5 and ING5-T152A were expressed in HEK293 cells as indicated and immunoprecipitated using mAb 7A11 and stained with mAb 3H5 for ING5-T152-P and with mAb 7A11 for ING5 (right panel).(F) Endogenous ING5 was immunoprecipitated from RIPA buffer lysates of HEK293 cells using a rabbit polyclonal serum (#3716), treated and analyzed as described in panel (D).

Phosphorylation by CDK2 is determined by the specificity of the kinase and the presence of cyclin binding motifs with the sequence RXL in the substrate [69]. Inspection of the ING5 amino acid sequence revealed two potential phosphorylation sites for CDKs (S57 and T152) as well as three RXL motifs (Fig 2A). Site-directed mutagenesis demonstrated that T152 was required for efficient phosphorylation in vitro by CDK2 (Fig 2B, left panel). Phosphorylation of ING5-T152A by cyclin E/CDK2 or cyclin A/CDK2 was substantially reduced in comparison to wild-type or ING5-S57A.

To address more directly whether T152 is phosphorylated, we generated mAbs against a peptide phosphorylated at T152. We selected mAb 3H5, which detected recombinant ING5 specifically when phosphorylated by cyclin E/CDK2 (Fig 2C). Together these findings suggest that ING5 is phosphorylated in vitro at T152 by CDK2.

### ING5 is phosphorylated at threonine 152 in cells

We were then interested to evaluate T152 phosphorylation in cells. ING5 and ING5-T152A were expressed in HEK293 cells, immunoprecipitated and analyzed for T152 phosphorylation using mAb 3H5. While ING5 was phosphorylated, the mutant protein was not (Fig 2D). Upon dephosphorylation of ING5 the epitope of the mAb 3H5 was lost, an effect that was antagonized by a phosphatase inhibitor (Fig 2D), supporting the conclusion that 3H5 specifically recognizes ING5-T152-P. To evaluate T152 phosphorylation further and to clarify that our mAb 7A11 recognizes also phosphorylated ING5, the proteins were immunoprecipitated and stained either with mAb 7A11 or with mAb 3H5 (Fig 2E). The findings demonstrated that T152-P is phosphorylated on endogenous ING5, as detected by mAb 3H5, and that mAb 7A11 recognized also phosphorylated ING5 (Fig 2E). As with overexpressed protein, the epitope was lost upon phosphatase treatment (Fig 2F).

**Fig 3:**
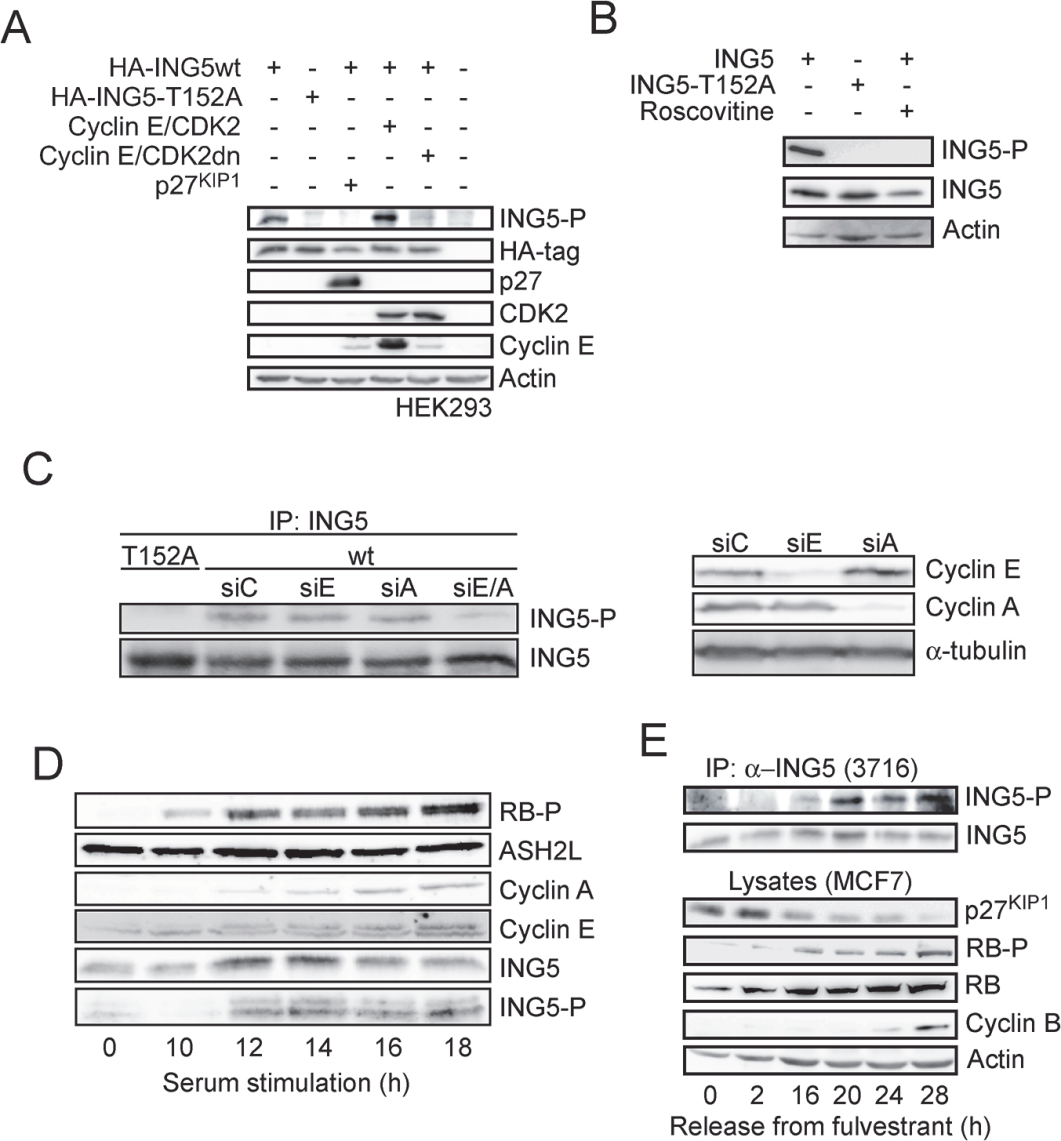
Phosphorylation of ING5 at T152 in cells. (A) The indicated proteins were expressed transiently in HEK293 cells. T152 phosphorylation was assessed using mAb 3H5. The other proteins were detected as detailed in the Material and Methods section. (B) ING5 or ING5-T152A were expressed transiently in HEK293 cells and treated with 25 μM roscovitine 2 h prior to harvesting. ING5 and ING5-P were analyzed using mAb 7A11 and 3H5, respectively. (C) ING5 or ING5-T152A were expressed transiently in HEK293 cells. The cells were then reseeded and transfected with control siRNA (siC) or siRNA targeting cyclin E (siE), cyclin A (siA) or both cyclins (siE/A). ING5 proteins were immunoprecipitated using mAB 7A11 and T152-P and total amounts of ING5 were analyzed using mAB 3H5 and 7A11, respectively. Functionality of the siRNAS were analyzed in total cell lysates (right panel). (D) The cells of the glioblastoma cell line T98A were arrested in G0 with 0.1% FCS. Cell cycle re-entry was stimulated by reseeding and stimulating with 10% FCS. The expression of the indicated proteins was analyzed at the indicated time points after G0 release. T152 phosphorylation was measured using mAb 3H5.(E) MCF7 cells were released from a fulvestrant block (1 μM, 72 h) by adding a 10-fold excess of tamoxifen. The expression of the indicated proteins was analyzed at the indicated time points after addition of tamoxifen. Endogenous ING5 was immunoprecipitated (pAb 3716) and analyzed using mAB 3H5 and 7A11.

Phosphorylation at T152 was enhanced in the presence of cyclin E/CDK2 but repressed when dnCDK2 or p27^KIP1^ were co-expressed (Fig 3A). Expanding on this finding, HEK293 cells transiently expressing ING5 were treated with roscovitine to inhibit CDK2 activity. This resulted in a complete loss of ING5 phosphorylation (Fig 3B). The knockdown of cyclin E and cyclin A also reduced T152-P (Fig 3C), further supporting the notion that cyclin E/CDK2 and cyclin A/CDK2 complexes phosphorylate T152 in cells. Additional evidence for cell cycle dependent T152 phosphorylation of ING5 was obtained in T98A glioblastoma cells. Arrested cells were re-stimulated with serum [64]. T152 phosphorylation was detected shortly after RB phosphorylation and when cyclin E and cyclin A were expressed (Fig 3D). Moreover we measured T152 phosphorylation of endogenous ING5 in MCF7 cells. These were arrested in G0/G1 by treatment with the estrogen receptor antagonist fulvestrant (ICI 182780). Re-entry of the cells into the cell cycle was stimulated with a 10-fold molar excess of tamoxifen [70], resulting in decreased expression of p27^KIP1^, increased phosphorylation of the retinoblastoma protein RB, and enhanced cyclin B expression (Fig 3E). Over the time course studied, the expression of ING5 did not vary, whereas the level of T152 phosphorylation increased parallel to the increase in RB phosphorylation (Fig 3E). Collectively these findings strongly argue for a CDK2-specific phosphorylation of ING5 at T152 in vitro and in cells.

### Phosphorylation at T152 does not affect subcellular localization

**Fig 4:**
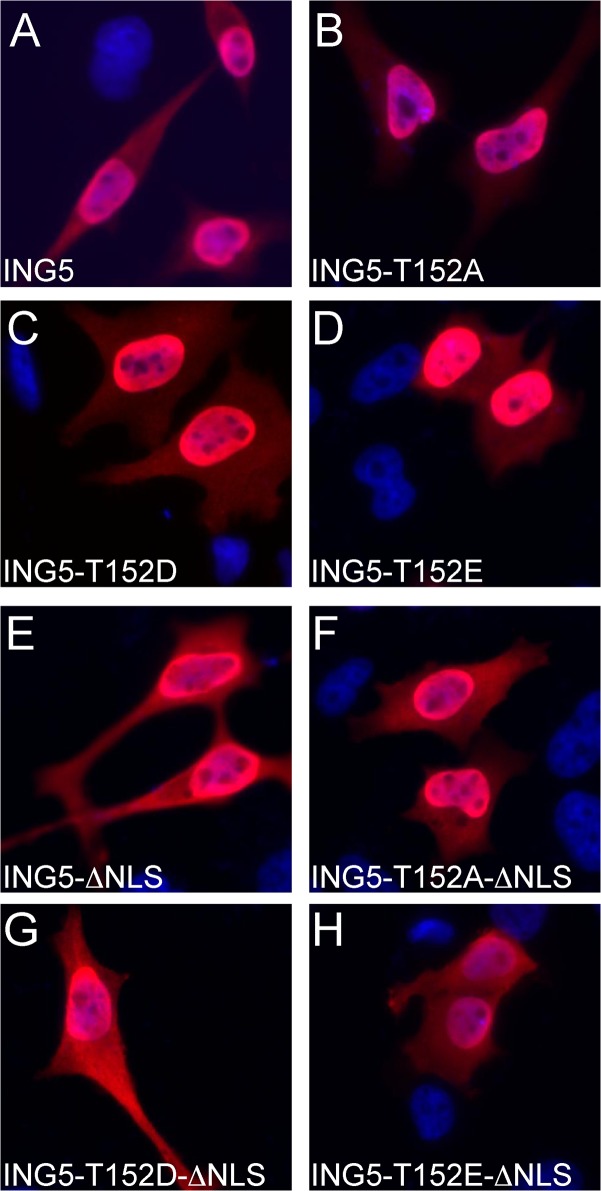
Phosphorylation of T152 does not affect subcellular localization of ING5. HA-tagged ING5 and ING5 phospho-site mutants, with and without a deleted C-terminal basic NLS (see Fig 2), were expressed in HeLa cells. ING5 was detected by indirect immunofluorescence staining using mAb 3F10.

ING5 possesses two NLS, a bipartite NLS from amino acids 128–171 and one other from amino acids 236–240 (Fig 2A) [37]. Thus T152 phosphorylation might affect subcellular localization by interfering with the bipartite NLS. ING5-T152A and two phospho-mimicking mutants ING5-T152D and ING5-T152E were expressed in HeLa cells. All proteins were nuclear (Fig 4A–4D). To address more specifically the bipartite NLS, the C-terminal NLS was deleted and the resulting proteins analyzed. All the proteins distributed equally between nuclear and cytosolic compartments (Fig 4E–4H). We have also expressed cyclin E/CDK2 together with ING5-ΔNLS and ING5-T152A-ΔNLS without observing any significant effect on the subcellular localization of the ING5 proteins (data not shown). This indicated that the C-terminal NLS is important to direct ING5 to the nucleus and that phosphorylation at T152 was unable to affect the function of the bipartite NLS. Thus we conclude that phosphorylation of T152 on its own is unlikely to explain the proposed cytosolic location of ING5 in some tumors [55,56,71], which might have been induced by oncogenic cyclin E/CDK2.

### Control of cell proliferation by ING5 is independent of p53

**Fig 5:**
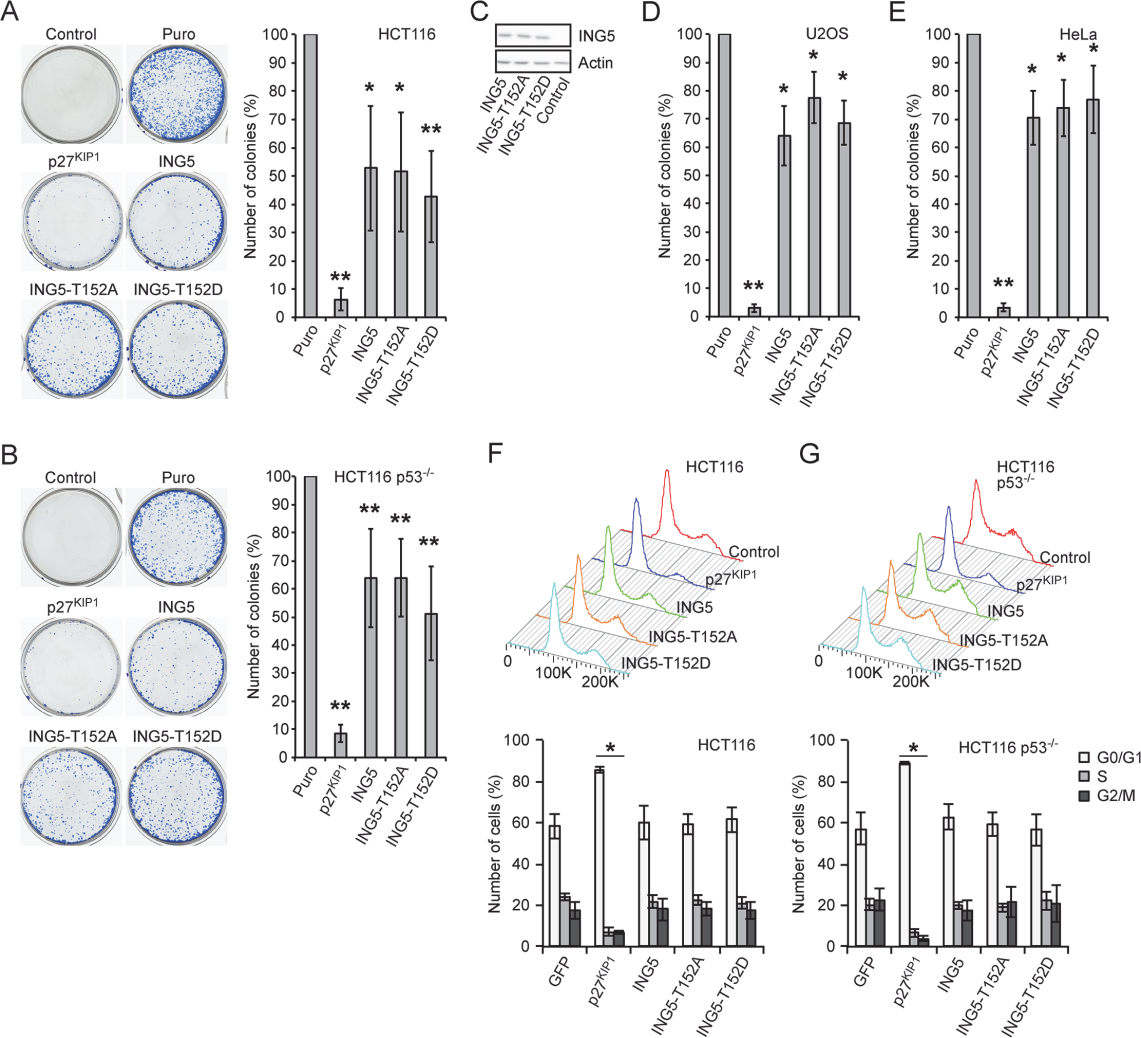
Overexpression of ING5 has only a minor effect on cell proliferation. (A) HCT116 cells were co-transfected with plasmids expressing the indicated proteins and a puromycin resistance plasmid. The cells were selected in puromycin and the growth of colonies evaluated 10 days after transfection by staining with methylene blue. Mean values and standard deviations of 5 independent experiments is depicted on the right.(B) As in panel (A) but with HCT116-p53^-/-^ cells. (C) ING5 and mutants were transiently expressed in HCT116 cells and the ING5 protein levels measured with mAb 7A11. Actin is analyzed for control. (D and E) As in panel (A) but with U2OS and HeLa cells. Three independent experiments were quantified for both cell lines. (F) HCT116 cells were co-transfected with plasmids expressing the indicated proteins and a plasmid encoding GFP-tubulin. Two days after transfection, the cells were stained with Vybrant and the cell cycle distribution of the transfected cells measured by FACS. Quantification of 3 experiments is shown at the bottom. (G) As in panel (F) but with HCT116-p53^-/-^ cells. * p<0.05, ** p<0.01.

It has been reported that ING5 binds to p53 and regulates its activity similar to other ING proteins [41]. We have also observed interaction of ING5 with p53, albeit weakly and rather variably (data not shown). Therefore we asked whether the postulated proliferation inhibitory effect of ING5 on cells is dependent on p53 and whether the phosphorylation of ING5 might have regulatory function. We overexpressed ING5 and T152 phospho-site mutants in HCT116 and HCT116-p53^-/-^ tumor cells (Fig 5A and 5B). ING5 and the mutants, which were expressed equally (Fig 5C), had only a small inhibitory effect in colony formation assays, with no consistent differences between the wild-type protein and the two mutants. Importantly we did not observe any difference between the parental HCT116 and the HCT116-p53^-/-^ cells (Fig 5A and 5B). For control p27^KIP1^, an efficient inhibitor of CDKs, was expressed and almost completely blocked cell proliferation as expected (Fig 5A and 5B). Additional colony formation assays were performed in U2OS cells, which are p53 positive, and in HeLa cells, which are functionally p53 negative due to the expression of E7 of human papilloma virus 18. ING5 and the phospho-site mutants had only small effects on the proliferation of these cells (Fig 5D and 5E). Consistent with the small effect on proliferation was that ING5 or the phospho-site mutants did not deregulate the cell cycle in HCT116 and HCT116-p53^-/-^ cells (Fig 5F and 5G). Thus, although ING5 interacted weakly with p53, we were unable to document effects on cell physiology that would have been consistent with regulation of endogenous p53. Similarly no effect on apoptosis was seen under these different settings, also an outcome that might have been expected in response to p53 activation (data not shown).

**Fig 6:**
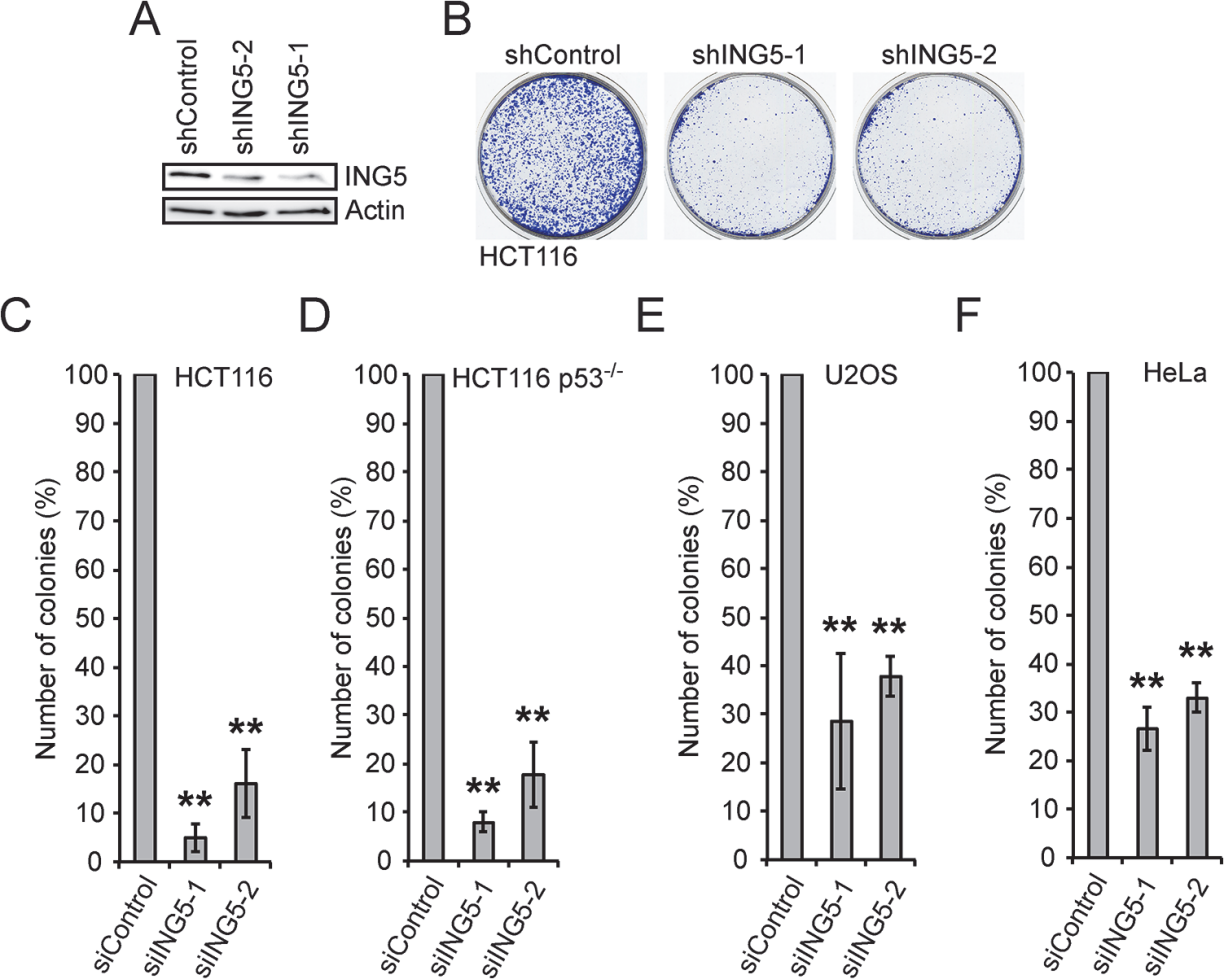
Knockdown of ING5 inhibits cell proliferation in tumor cells independent of p53. (A) HEK293 cells were transfected with the indicated pSuper constructs. The expression of ING5 was analyzed using mAb 7A11. Actin is shown for control. (B) HCT116 cells were co-transfected with plasmids expressing the indicated shRNAs and a puromycin resistance plasmid. The puromycin selected cells were evaluated 10 days after transfection by staining with methylene blue. (C-F) Quantification of 3 independent experiments with HCT116 (C), HCT116-p53^-/-^ (D), U2OS (E) and HeLa (F) cells. ** p<0.01.

The lack of effects in response to ING5 overexpression was unexpected, particularly the apparent irrelevance of p53. In contrast to overexpression, previous studies had suggested that the knockdown of ING5 resulted in the accumulation of cells in S phase [45]. Therefore we tested whether ING5 was necessary for cell proliferation dependent on the p53 status. Two different shRNA constructs were selected for their ability to repress endogenous ING5, with shING5-1 being somewhat more efficient than shING5-2 (Fig 6A). Both efficiently repressed cell proliferation in HCT116 as well as in HCT116-p53^-/-^ cells, i.e. irrespective of the presence or absence of p53 (Fig 6B–6D). Similarly the knockdown of ING5 repressed U2OS and HeLa cell proliferation (Fig 6E and 6F). Thus the findings suggest that in these tumor cells ING5 expression is essential for proliferation. This is consistent with the observation in epidermal stem cells, in which ING5 is important for cell proliferation [63], and with the role of ING5 in replication of MCF7 tumor cells [45].

### Knockdown of ING5 induces cell death independent of p53

**Fig 7:**
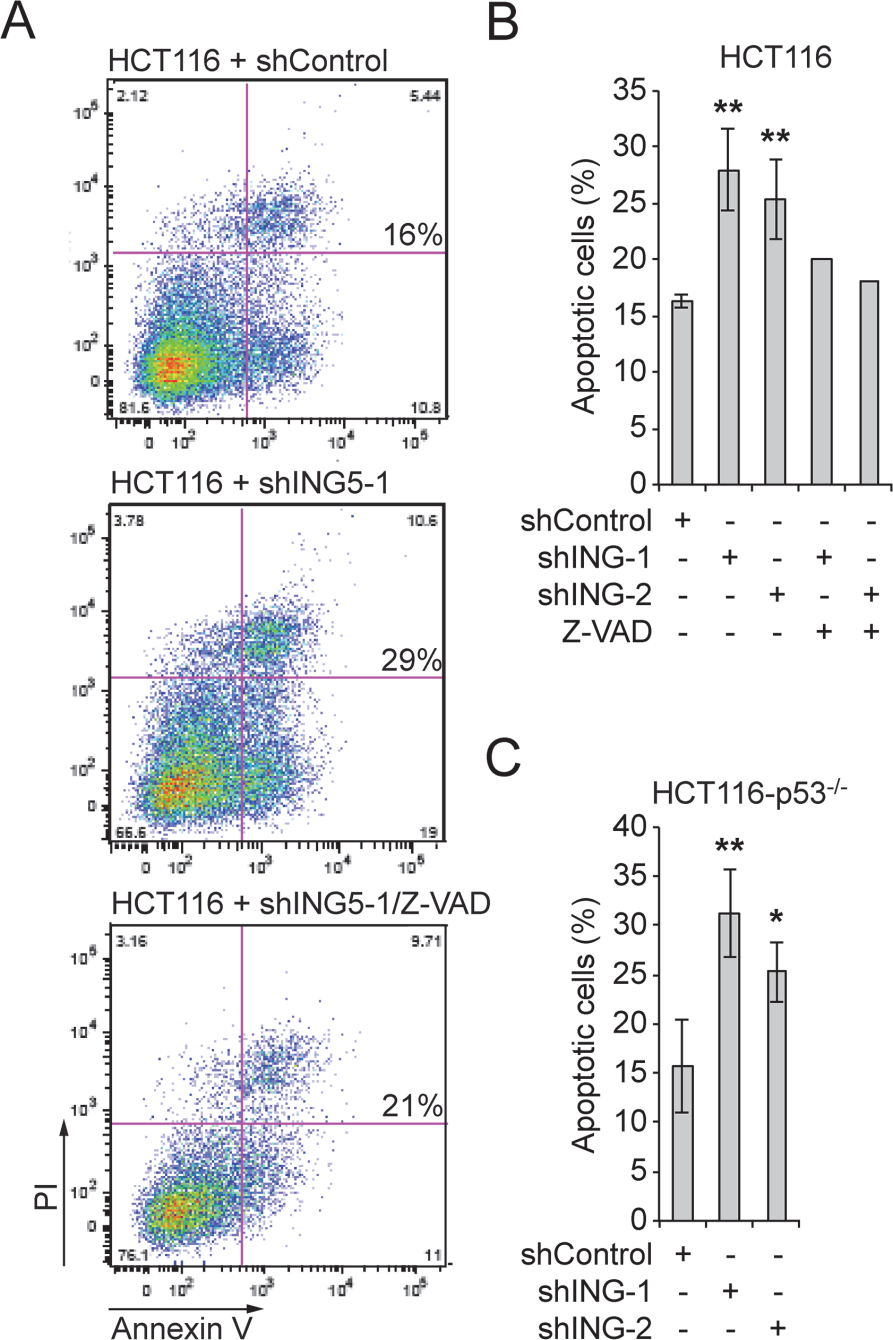
Knockdown of ING5 induces apoptosis independent of p53. (A) HCT116 cells were co-transfected with the indicated pSuper constructs and a plasmid encoding GFP-tubulin. The cells were treated with or without Z-VAD. The cells were stained for annexin V and with propidium iodide and analyzed by FACS 48 h after transfection. GFP-positive cells were gated. (B) Quantification of experiments with HCT116 cells. Mean values and standard deviations of 3 independent experiments are displayed for the shRNA experiments. For Z-VAD mean values of 2 experiments are shown.(C) Quantification of experiments as in panel (B) but with HCT116-p53^-/-^ cells. * p<0.05, ** p<0.01.

The consequences of ING5 knockdown on colony formation might be due to cell cycle arrest in S phase as suggested previously [45]. Indeed we also observed a minor accumulation of cells in S phase (data not shown). However we also noted in our experiments that the number of viable cells decreased over time. Therefore we measured the appearance of annexin V positive cells as an indication of apoptosis. Within 48 h after transfection of shING5 expressing plasmids, we observed a significant increase in annexin V positive cells (Fig 7). Induction of apoptosis in HCT116 cells was blocked by Z-VAD, a broad-spectrum caspase inhibitor (Fig 7A). The effects on induction of apoptosis were comparable in HCT116 and HCT116-p53^-/-^ cells (Fig 7B and 7C). Thus our findings suggest that ING5 affects proliferation, S phase progression, and apoptosis independent of p53.

## Discussion

We report that ING5 is phosphorylated in a cell cycle dependent manner by CDK2 at T152 (Figs 1 and 3). Although this phosphorylation site is part of the bipartite NLS of ING5, a mutant that cannot be phosphorylated, phospho-mimicking mutants, and the co-expression of cyclin E/CDK2 did not affect subcellular localization when measured under steady-state conditions (Fig 4). Our further analysis indicates that ING5 and ING5 phospho-site mutants have a minor effect on cell proliferation and that this is independent of the presence of p53 (Fig 5). Similarly the knockdown of ING5, which is strongly growth inhibitory at least in part by inducing apoptosis, is independent of p53 (Figs 6 and 7).

Although ING proteins are associated with different post-translational modifications (PTM), including their ability to recognize specific histone modifications and modulating the PTMs of p53 [41,42,43,44,72,73,74,75], we know relatively little about PTMs of ING proteins and their functional relevance. But it is to be expected that these proteins are regulated in multiple ways to perform their functions in controlling gene transcription and replication and ultimately affect cell behavior. For ING1b it was demonstrated that it is phosphorylated at S126 by CHK1 in response to DNA damage [76]. This phosphorylation stabilizes the protein. We have considered a role of T152 phosphorylation for regulating ING5 stability but were unable to obtain supportive evidence (data not shown). Enhanced protein stability of ING1b-S126-P might be relevant to mediate stress response through p53 and is important to down-regulate cyclin B1, thus interfering with proliferation. ING1b is also phosphorylated at S199, which generates a binding site for 14-3-3 proteins, promoting cytoplasmic localization [77]. S199-P interferes with the ability of ING1b to stimulate p21^WAF1^ expression, thus phosphorylation at S126 and S199 appear to have opposing roles in controlling ING1b function. Unlike shown for ING1b, our experiments did not uncover a role of ING5-T152 phosphorylation in controlling subcellular localization or cell proliferation, although the latter was expected with the described roles of both ING5 and cyclin E/CDK2 in replication (see Introduction). Cyclin E/CDK2 is also associated with transcription and controls for example the oncoprotein MYC [78]. Thus it is possible that ING5, potentially as part of HAT complexes, participates in selective gene regulation, an aspect that needs to be further analyzed.

ING proteins have attracted interest because of their involvement in cancer and because of their effects on the tumor suppressor protein p53 [35,36,37]. ING5 has also been suggested to regulate p53 [41,59]. Moreover down-regulation or cytoplasmic re-localization of ING5 has been observed in a number of tumors, suggesting that ING5 might function as a tumor suppressor [52,53,54,55,56,57,71]. In contrast to these studies, which are largely correlative, several studies suggest that ING5 may also have pro-tumorigenic functions. Tamoxifen resistance in breast cancer is a major therapeutic problem. Because the knockdown of ING5 in MCF7 cells promotes tamoxifen sensitivity, ING5 expression may be associated with tumor progression [58]. Moreover ING5 expression correlates with maintaining proliferation in epidermal stem cells [63]. These findings are consistent with the inhibition of proliferation in HCT116, U2OS and HeLa cells, independent of the p53 status, in response to the knockdown of ING5 (Fig 6). Its primary consequence is apoptosis in HCT116 cells (Fig 7), while we did not observe significant effects on cell cycle distribution, unlike reported previously [45]. This study aimed at revealing the pathophysiological mechanisms of ING5 action in the regulation of proliferation. The most pertinent assumptions were tested with the experiments presented here, in particular for the role of phosphorylation of ING5, but for the clarification of the functional importance of this PTM further work is needed. The knowledge obtained here will be the basis for further studies as it will now be interesting to define the targets of ING5 that affect apoptosis and cell proliferation and to understand in more detail what the role of CDK2-dependent phosphorylation might be.
