# Expression of Concern: ING5 is phosphorylated by CDK2 and controls cell proliferation independently of p53

**DOI:** 10.1371/journal.pone.0351194

**Published:** 2026-06-09

**Authors:** The PLOS One Editors

After this article [1] was published, several concerns were raised. Specifically,

- The shING5-1 and shING5-2 panels in Fig 6B appear to overlap.
- Figs 5-7 report the results of statistical analyses, however the article [1] does not describe the statistical tests used.
- The original quantitative data underlying the figures have not been provided, contrary to the Data Availability statement.

Co-corresponding author JV provided an updated version of Fig 6 with replacement images for all panels in Fig 6B from replicate experiments conducted at the time of the original experiments. The quantified data from Fig 6B presented in Fig 6C are updated to include the replacement panels in the updated Fig 6B, such that the updated Fig 6C shows the mean and standard deviation of four measurements. With the updated Fig 6, PLOS considers this concern resolved.

Regarding the statistical methodology used in [1], co-corresponding author JV stated that they were unable to identify the statistical analyses used in this article. As the statistical methods used cannot be confirmed, the *PLOS One* Editors advise readers to interpret the statistical results in Fig 5 and 7 with caution.

Co-corresponding author JV provided the data underlying Figs 1 and 4, and partial underlying data for Figs 2-3 and 5–7 (S1–S9 Files). They stated the remainder of the underlying data are no longer available. As all original raw data files supporting the article’s results have not been made available, this article [1] does not comply with the PLOS Data Availability policy in place at the time this article was submitted.

During editorial review, it was noted that the underlying data provided for Figs 7B and 7C do not appear to match the published figures. Specifically:

- In Fig 7B, the shING-1 + Z-VAD- mean appears higher than the shING-2 + Z-VAD- mean, whereas in the underlying data (S7 File), the shING5−1 mean appears lower than the shING5−2 mean.
- In Fig 7C, the shING-1 mean appears higher than the shING-2 mean, whereas in the underlying data (S7 File), the shING5−1 mean appears lower than the shING5−2 mean.

JV acknowledged this discrepancy and stated that as all original data are not available, they are unable to explain the cause of the error. PLOS remains concerned for the results presented in Figs 7B and 7C.

In light of the above unresolved concerns, the *PLOS One* Editors issue this Expression of Concern and advise that the results presented in this article be interpreted with caution.

**Fig 6:**
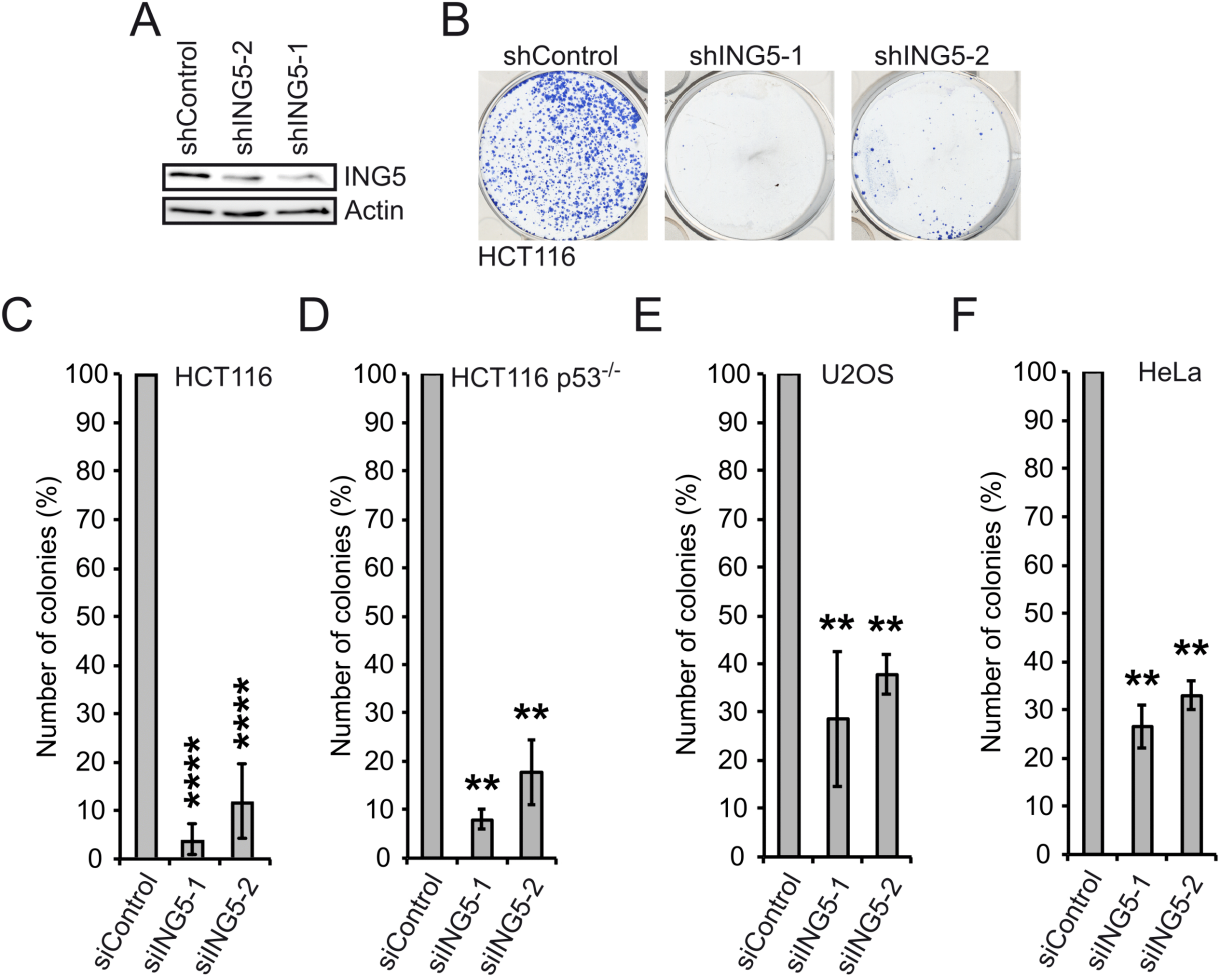
Knockdown of ING5 inhibits cell proliferation in tumor cells independent of p53. **(A)** HEK293 cells were transfected with the indicated pSuper constructs. The expression of ING5 was analyzed using mAb 7A11. Actin is shown for control. **(B)** HCT116 cells were co-transfected with plasmids expressing the indicated shRNAs and a puromycin resistance plasmid. The puromycin selected cells were evaluated 10 days after transfection by staining with methylene blue. **(C-F)** Quantification of 2-4 independent experiments with HCT116 **(C)**, HCT116-p53^-/-^ **(D)**, U2OS (E) and HeLa (F) cells. ** p < 0.01, ****p < 0.0001. For panel C, ordinary one-way ANOVA followed by Tukey’s multiple comparison of four independent experiments were used.

## Supporting information

S1 File Quantitative data and original blots underlying Fig 1. (ZIP)

S2 File Quantitative data and original blots underlying Fig 4. (ZIP)

S3 File Quantitative data and original blots partially underlying Fig 2. (ZIP)

S4 File Quantitative data and original blots partially underlying Fig 3. (ZIP)

S5 File Quantitative data and original blots partially underlying Fig 5A. (ZIP)

S6 File Quantitative data and original blots partially underlying Fig 5B. (ZIP)

S7 File Quantitative data and original blots partially underlying Figs 5F-5G. (ZIP)

S8 File Quantitative data and original blots partially underlying Fig 6. (ZIP)

S9 File Quantitative data and original blots partially underlying Fig 7. (ZIP)
