# Supplementary figures and images for: Expression of Concern: ING5 is phosphorylated by CDK2 and controls cell proliferation independently of p53
Source: PLoS One. 2026 Jun 9;21(6):e0351194. doi: 10.1371/journal.pone.0351194 (PMC13249149; doi:10.1371/journal.pone.0351194)

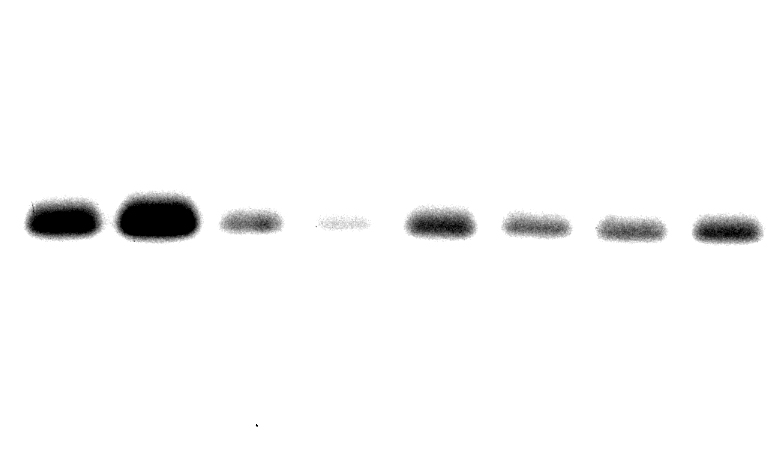

Supplement: S1 File — (ZIP) [file pone.0351194.s001.zip › S1 File/Fig1B/Fig 1B_1_InvivoP nur Banden Kopie.jpg]

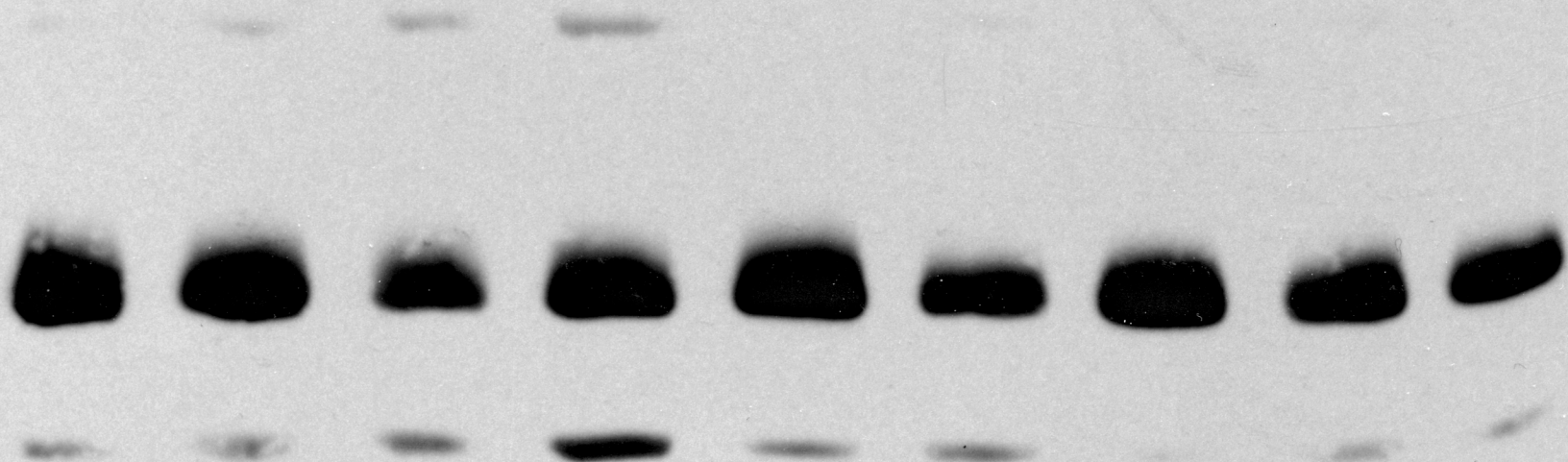

Supplement: S1 File — (ZIP) [file pone.0351194.s001.zip › S1 File/Fig1B/Fig 1B_2_Scan WB Figure 3b.pdf]

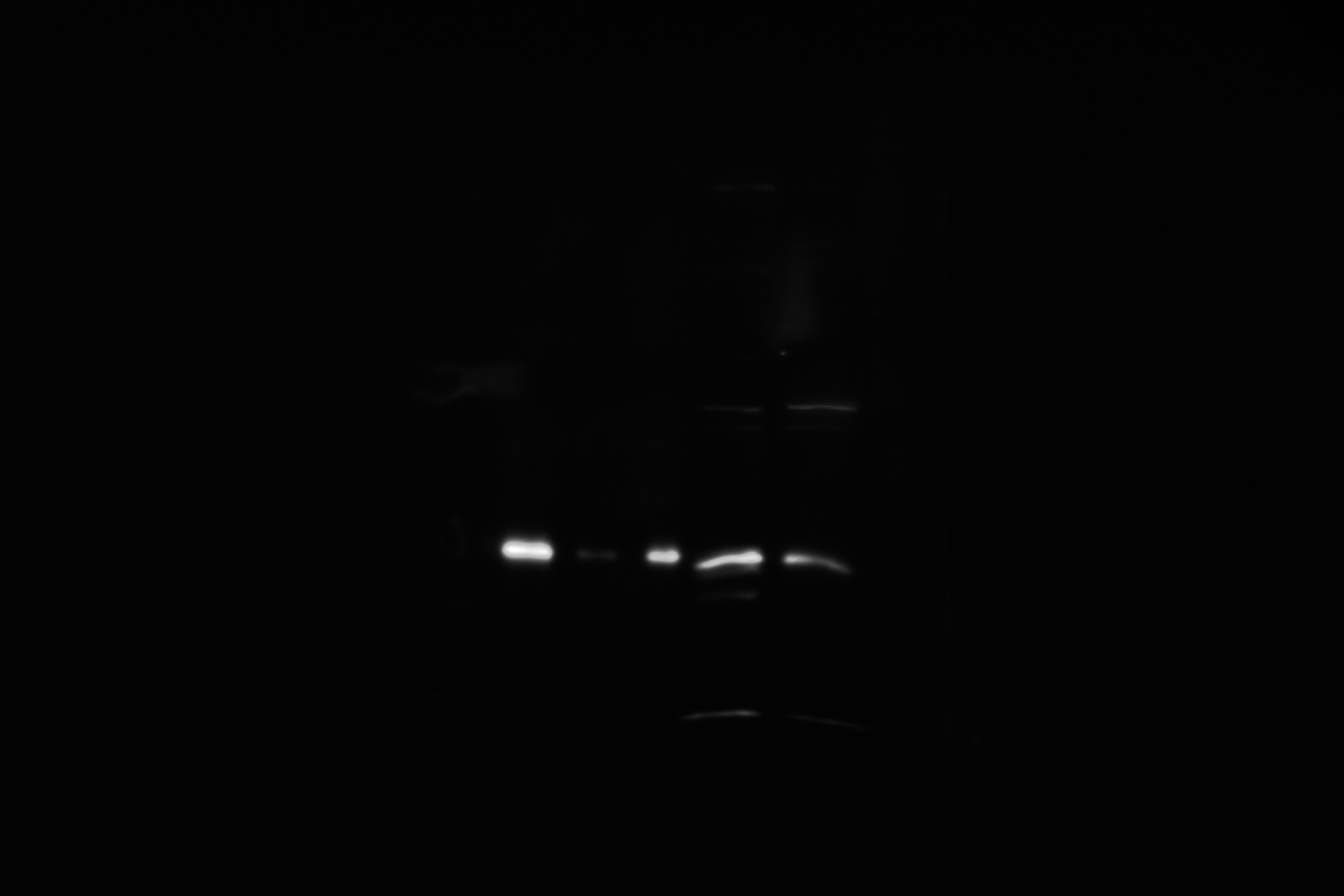

Supplement: S1 File — (ZIP) [file pone.0351194.s001.zip › S1 File/Fig1C/Fig 1C_1_a-Jas Char. 1min Kopie.tif]

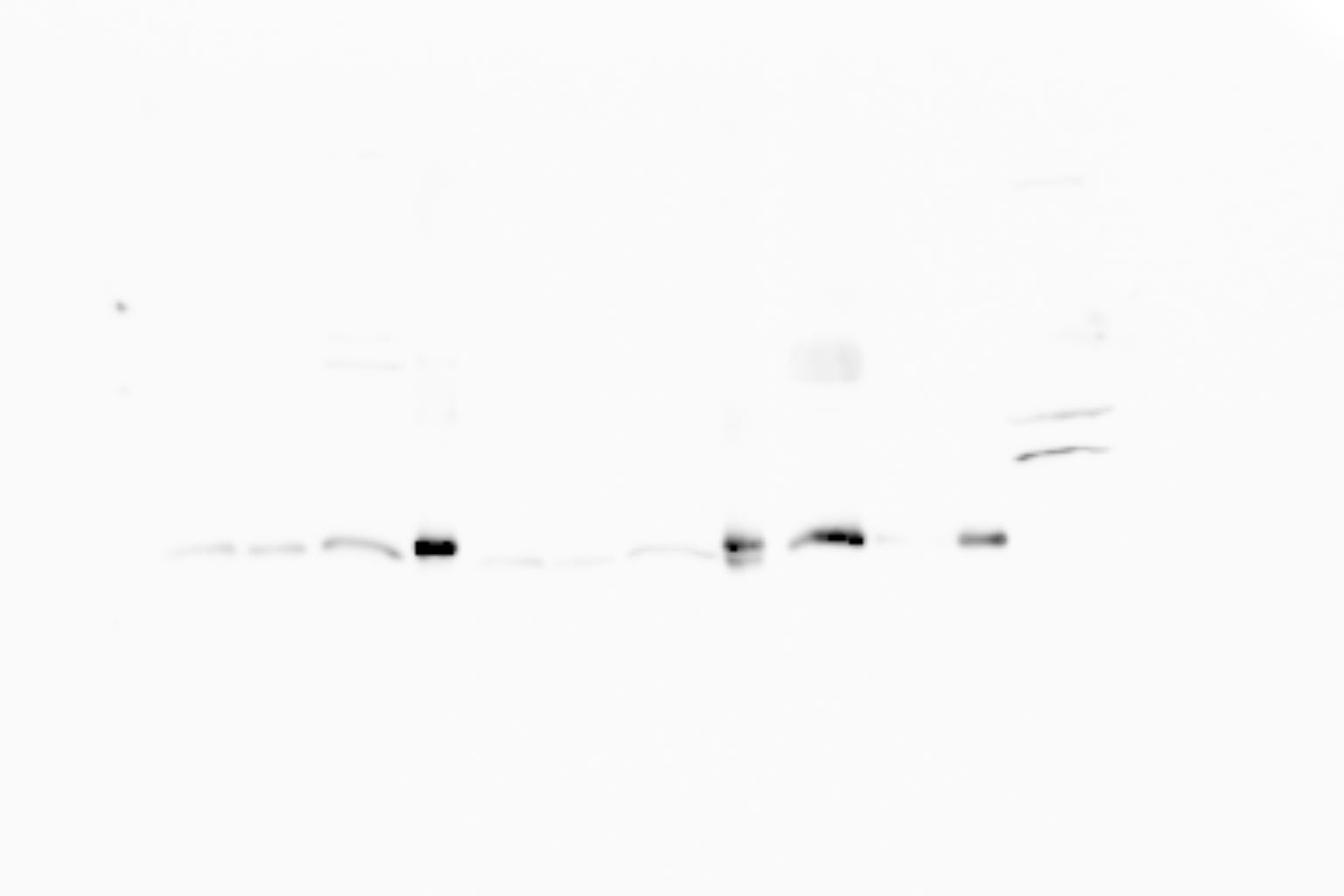

Supplement: S1 File — (ZIP) [file pone.0351194.s001.zip › S1 File/Fig1C/Fig 1C_2_bl.4,5,6 30sec a-HA Kopie.tif]

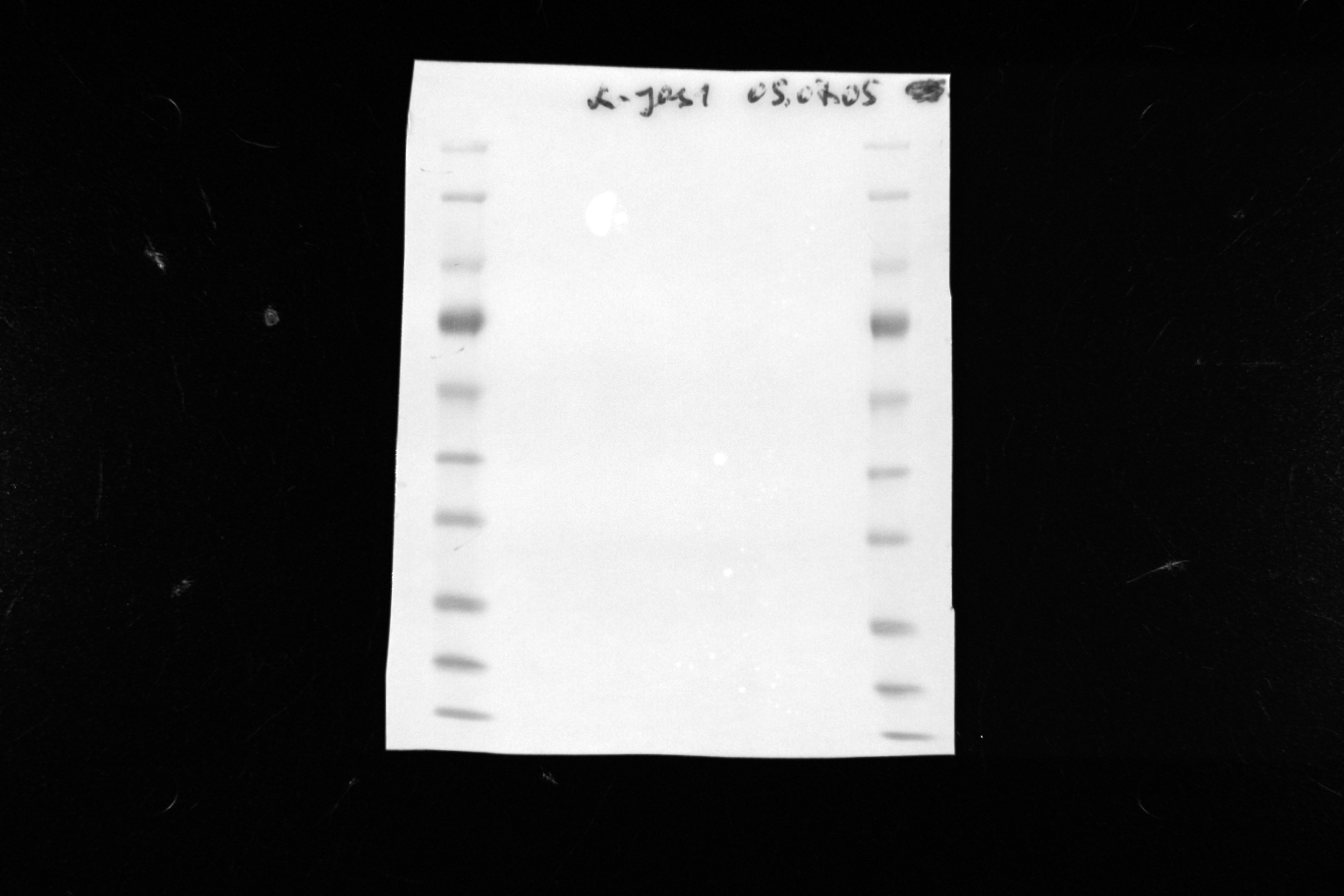

Supplement: S1 File — (ZIP) [file pone.0351194.s001.zip › S1 File/Fig1C/V_a-Jas Char. 1min Kopie.tif]

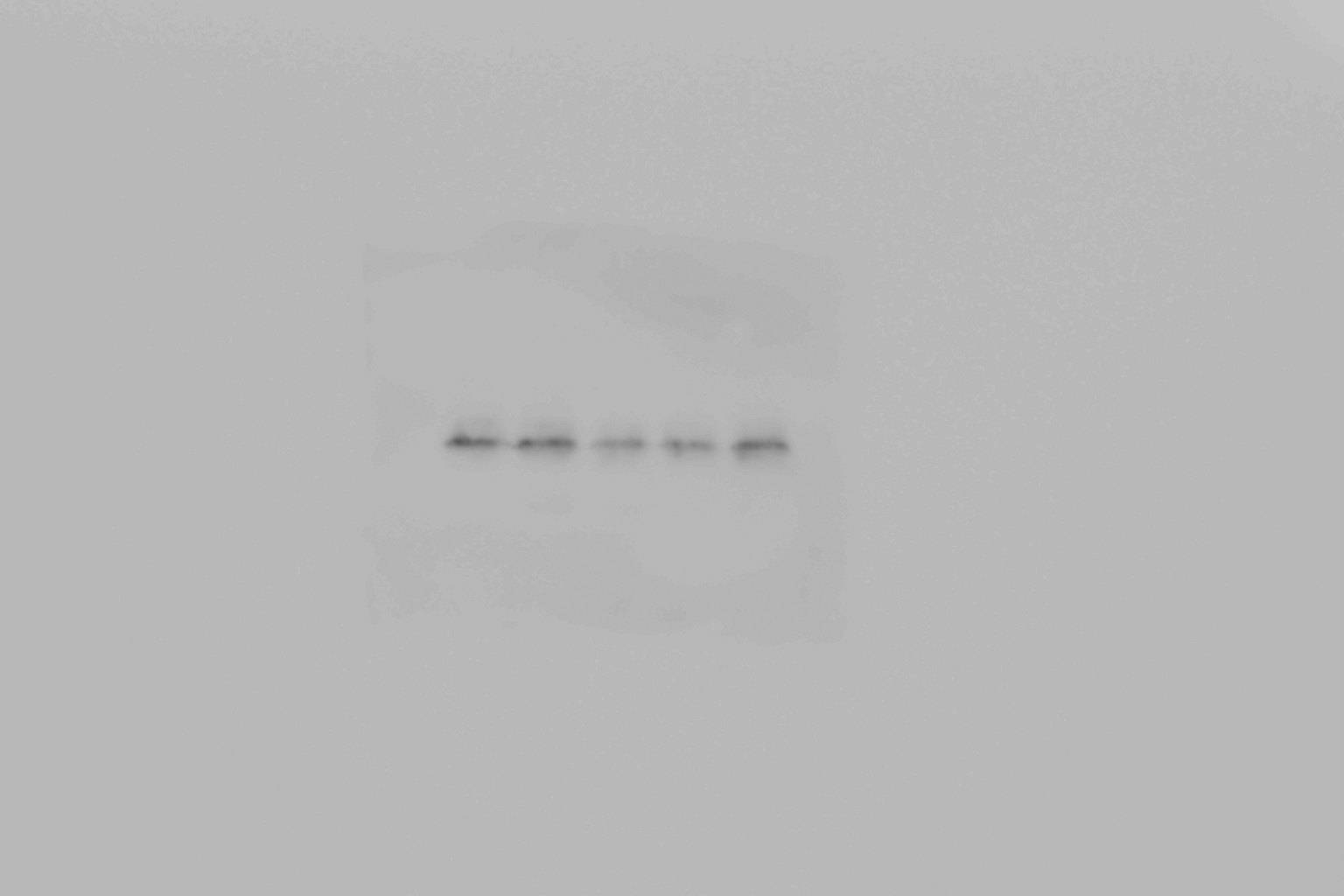

Supplement: S1 File — (ZIP) [file pone.0351194.s001.zip › S1 File/Fig1D/Fig 1D_2_WB 32P lab ING5 low Kopie.tif]

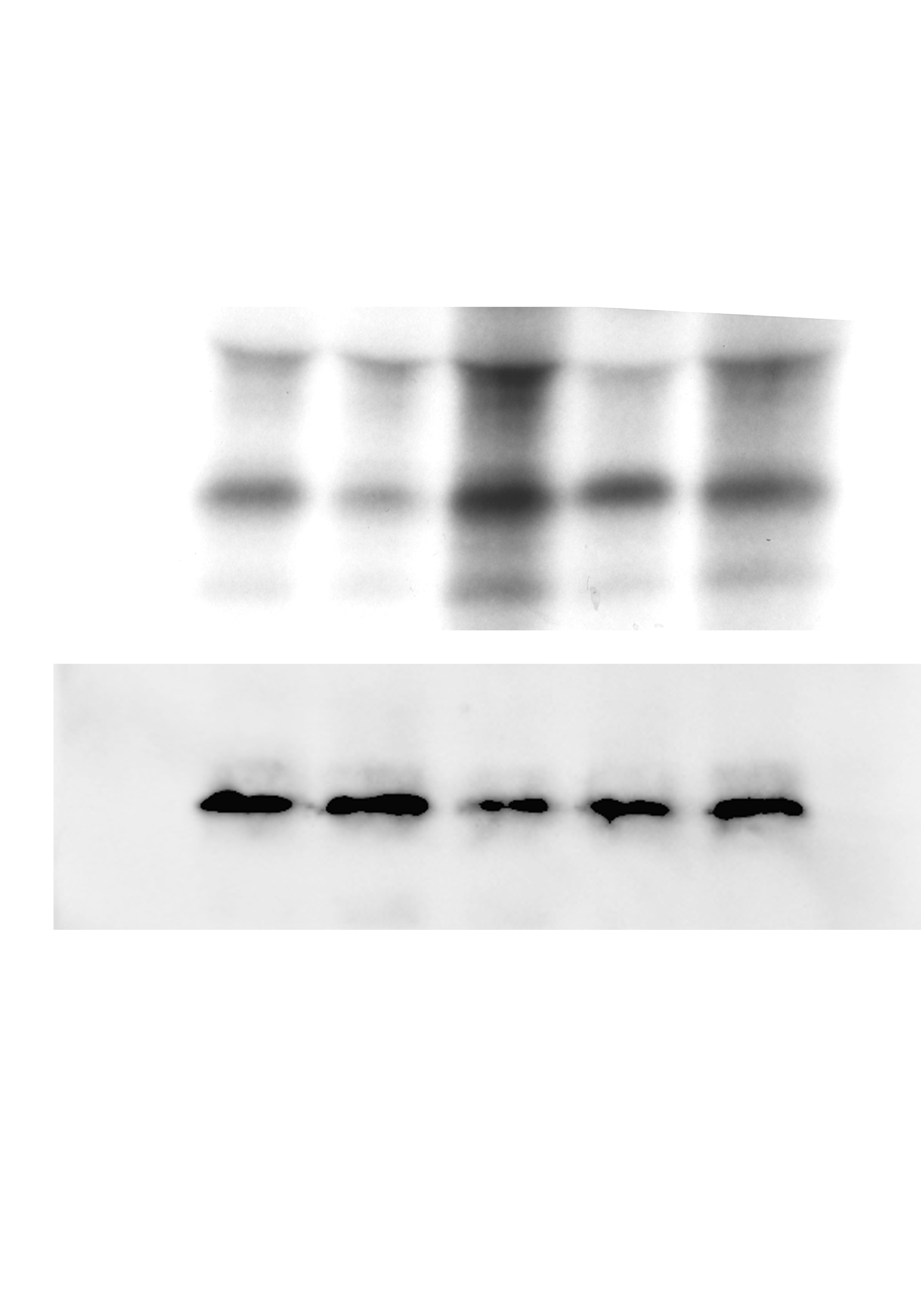

Supplement: S1 File — (ZIP) [file pone.0351194.s001.zip › S1 File/Fig1D/Fig 1D_ING5 invivo lab + WBver2 Kopie 2.jpg]

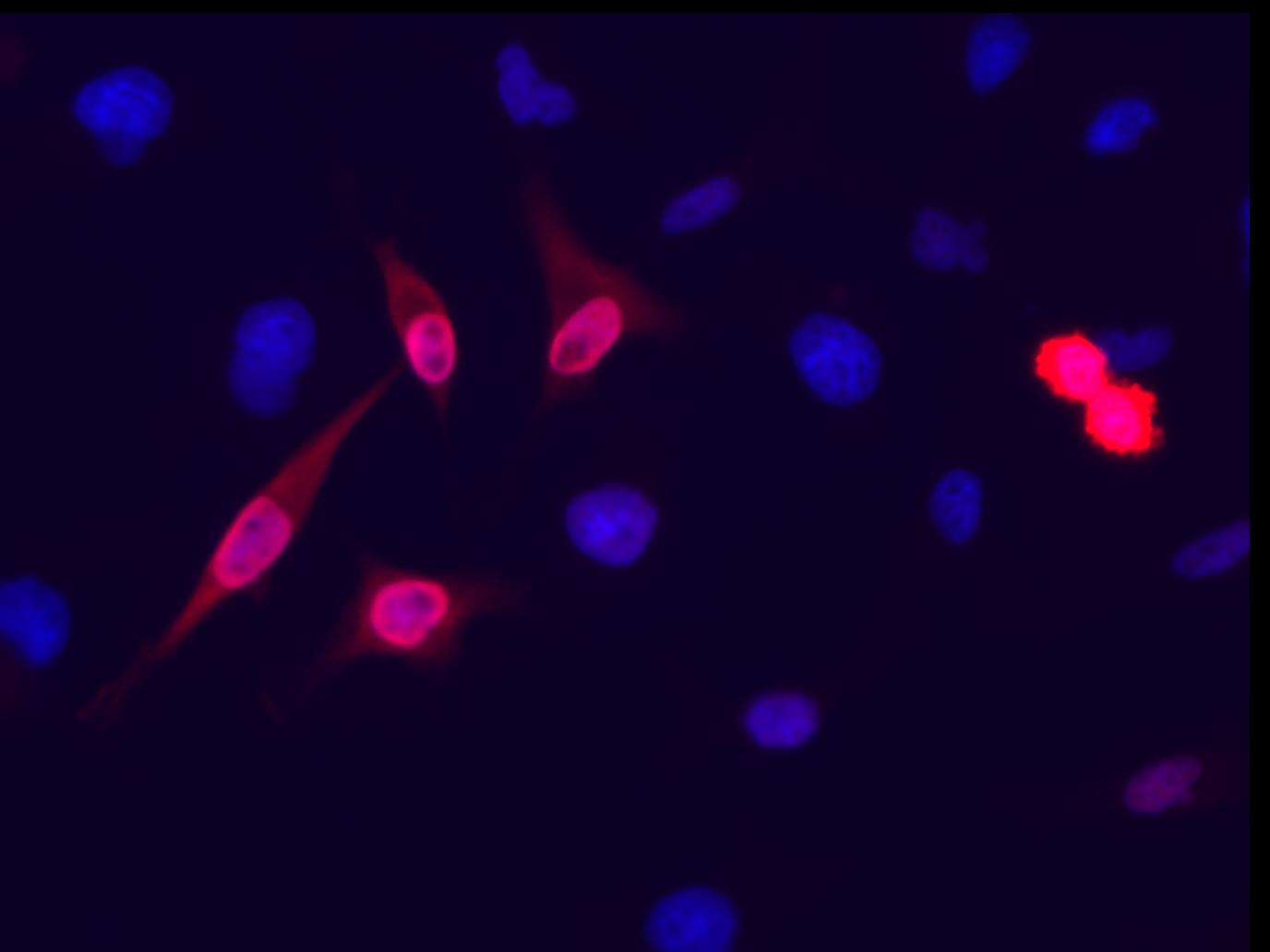

Supplement: S2 File — (ZIP) [file pone.0351194.s002.zip › S2 File/4A.jpg]

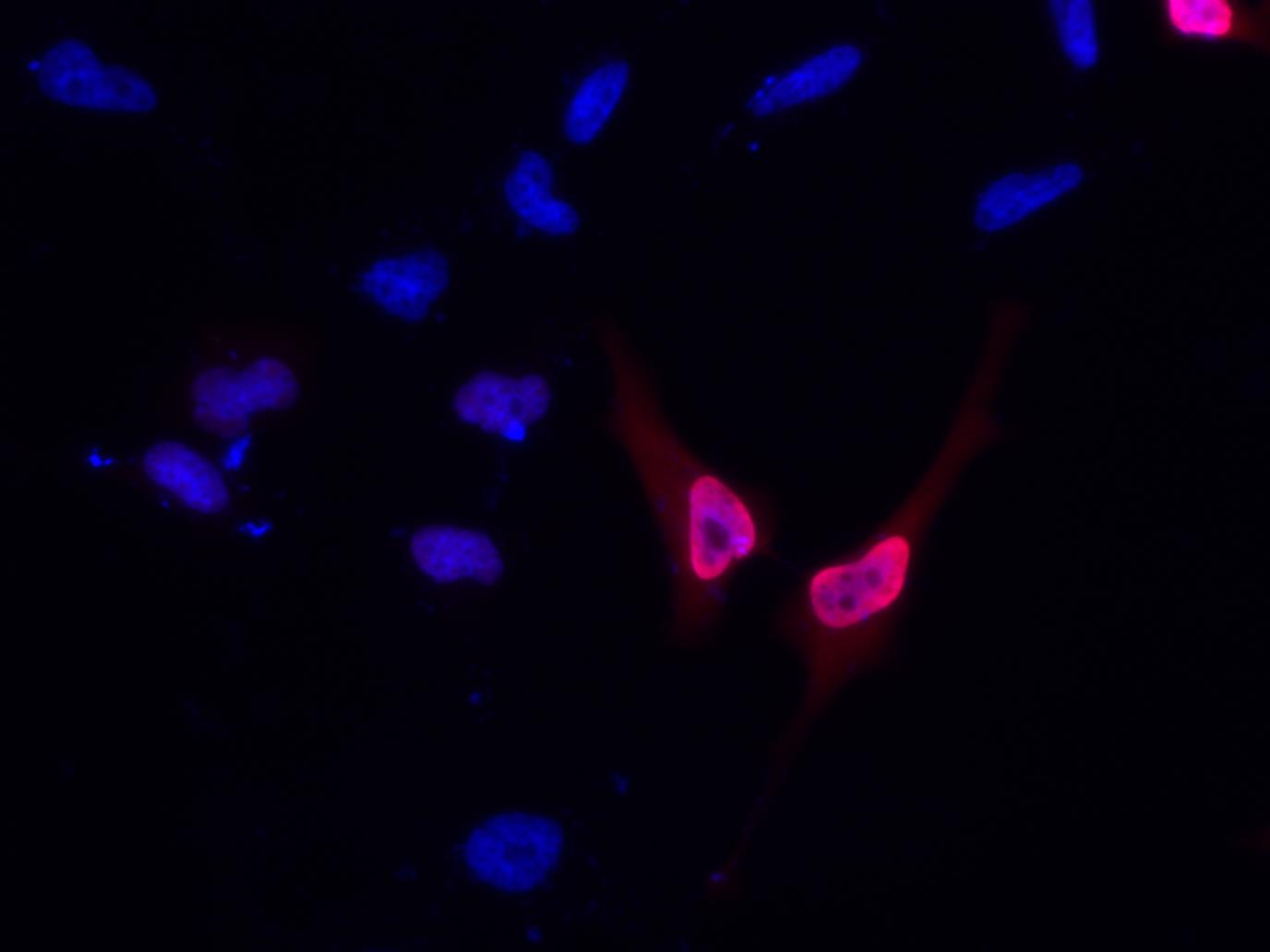

Supplement: S2 File — (ZIP) [file pone.0351194.s002.zip › S2 File/4B.jpg]

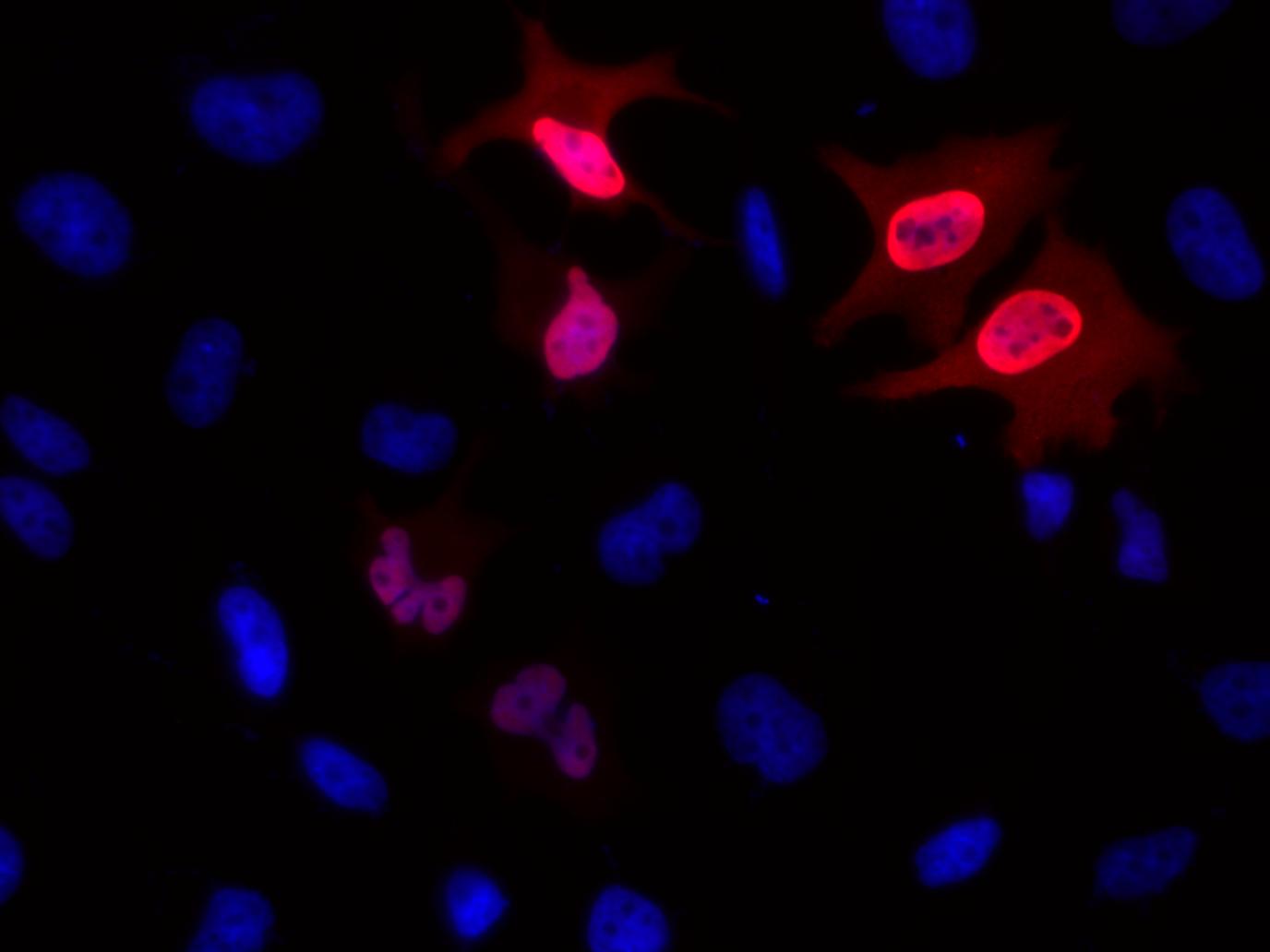

Supplement: S2 File — (ZIP) [file pone.0351194.s002.zip › S2 File/4C.jpg]

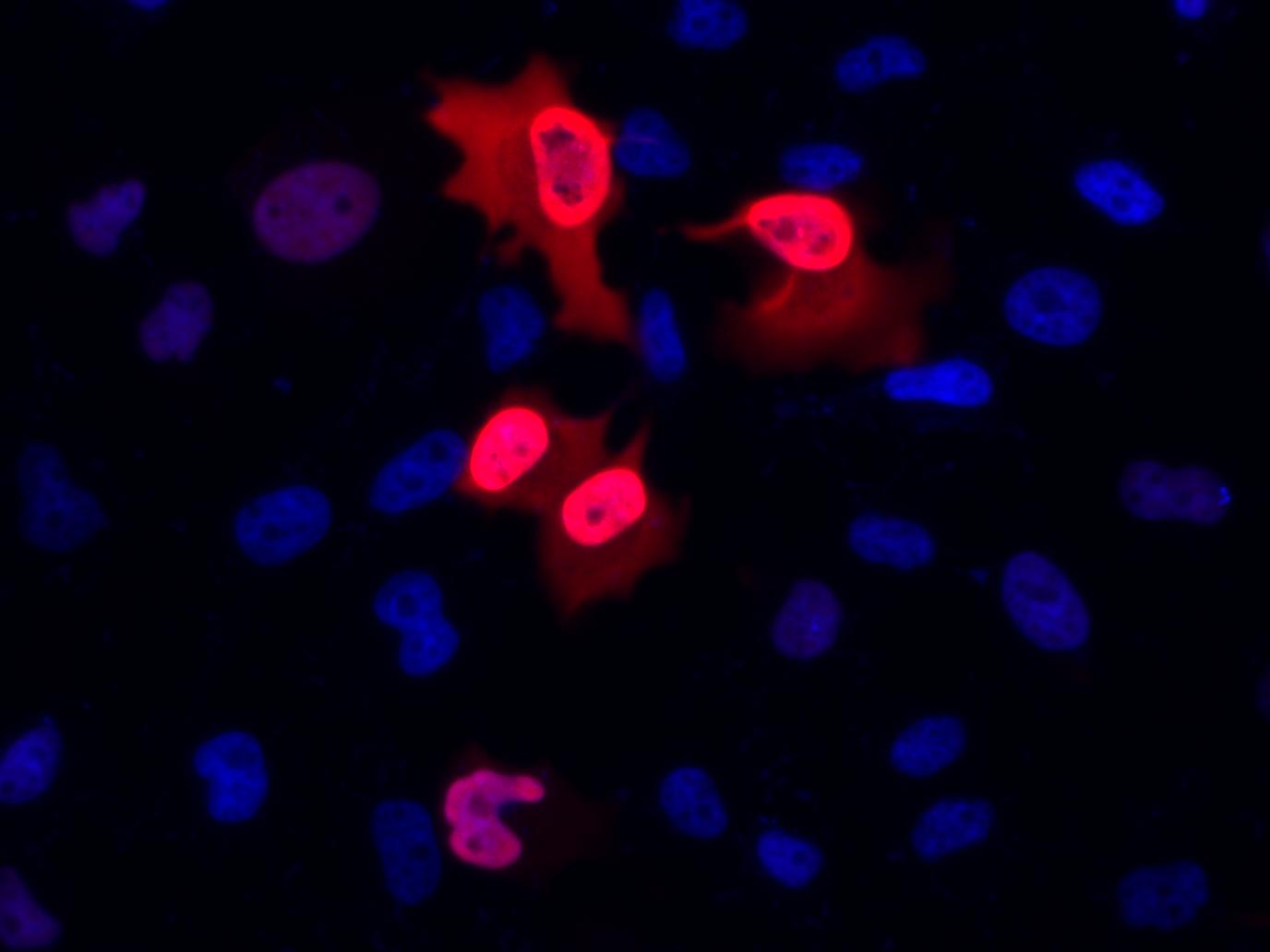

Supplement: S2 File — (ZIP) [file pone.0351194.s002.zip › S2 File/4D.jpg]

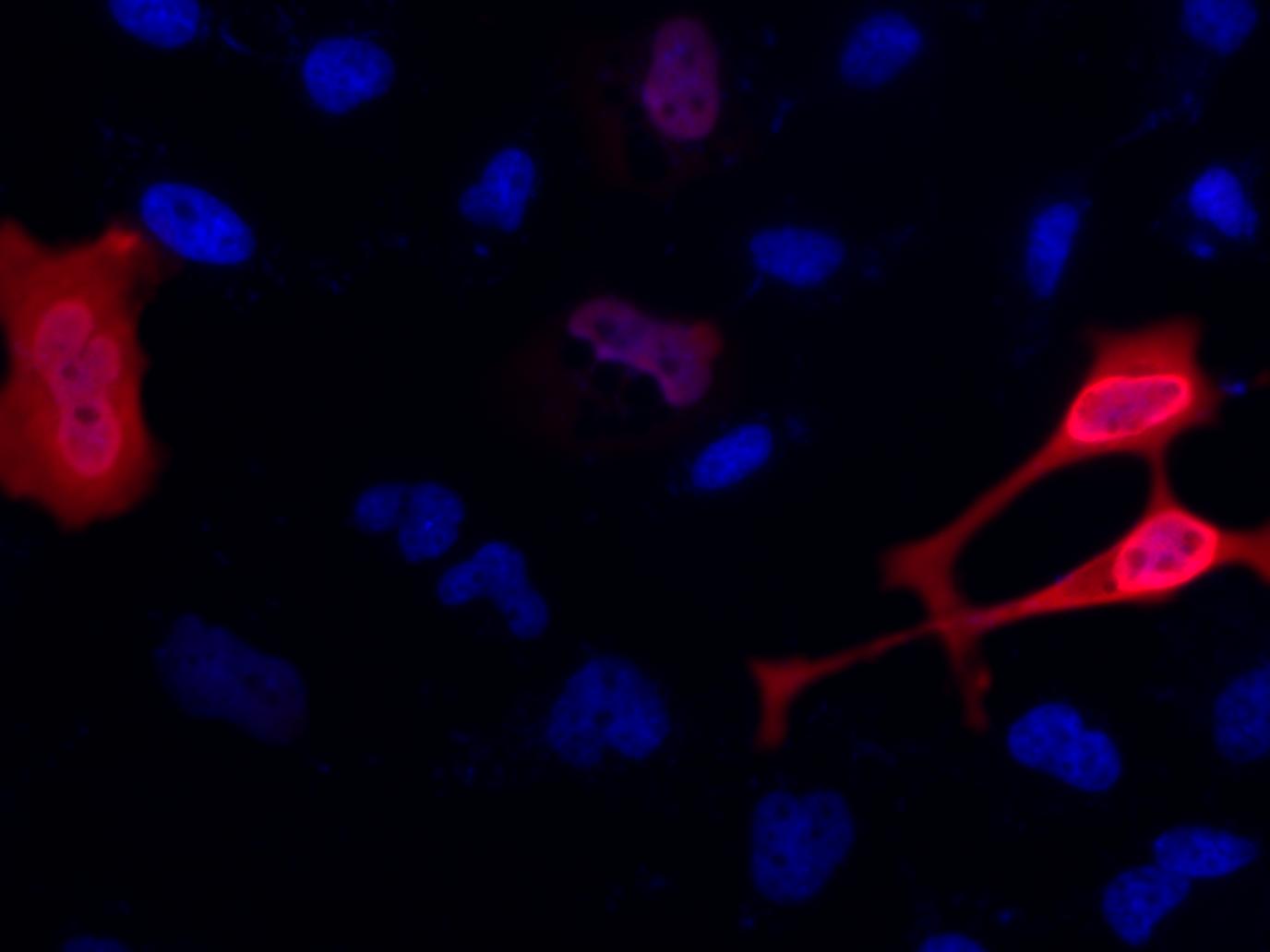

Supplement: S2 File — (ZIP) [file pone.0351194.s002.zip › S2 File/4E.jpg]

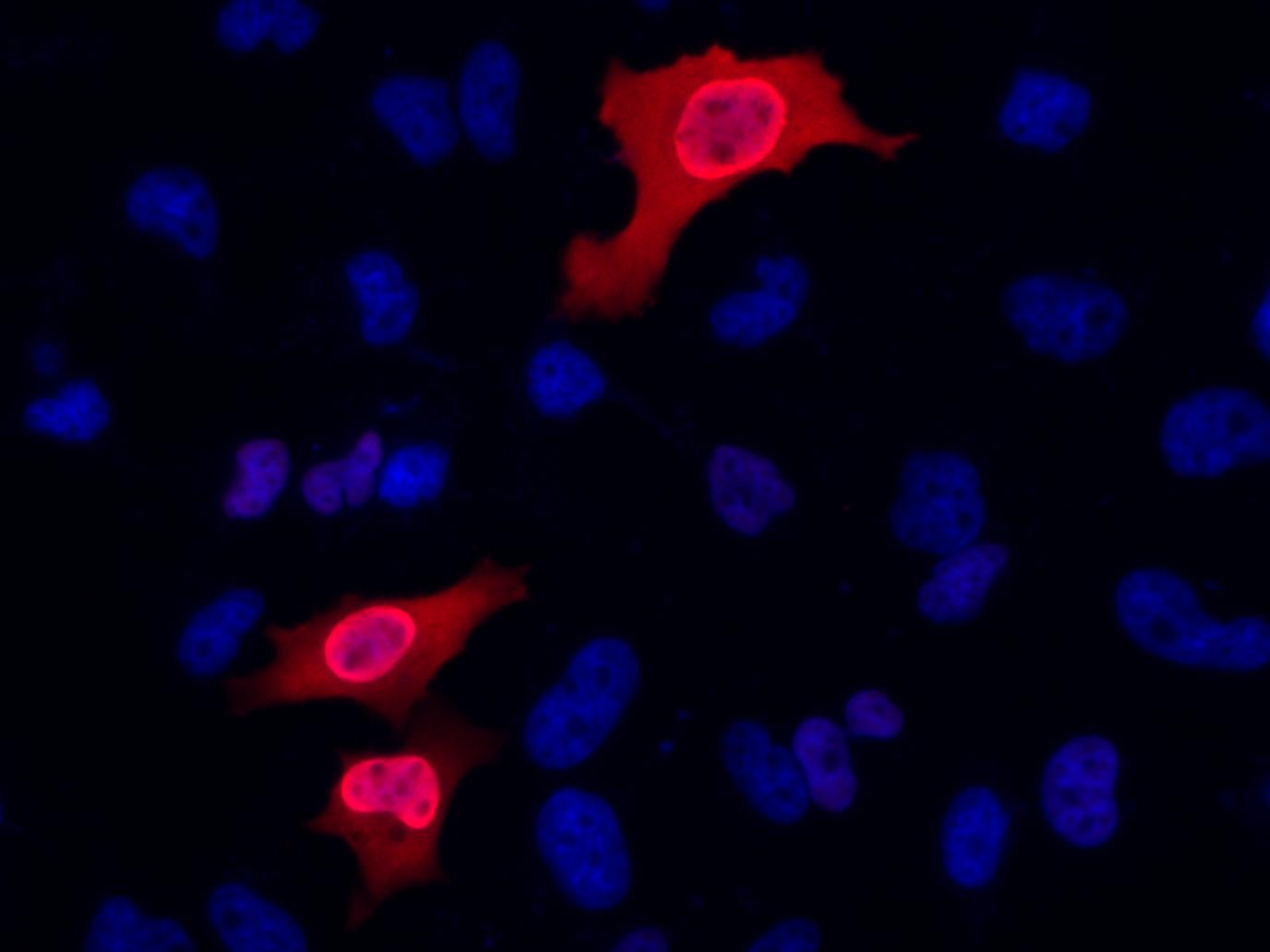

Supplement: S2 File — (ZIP) [file pone.0351194.s002.zip › S2 File/4F.jpg]

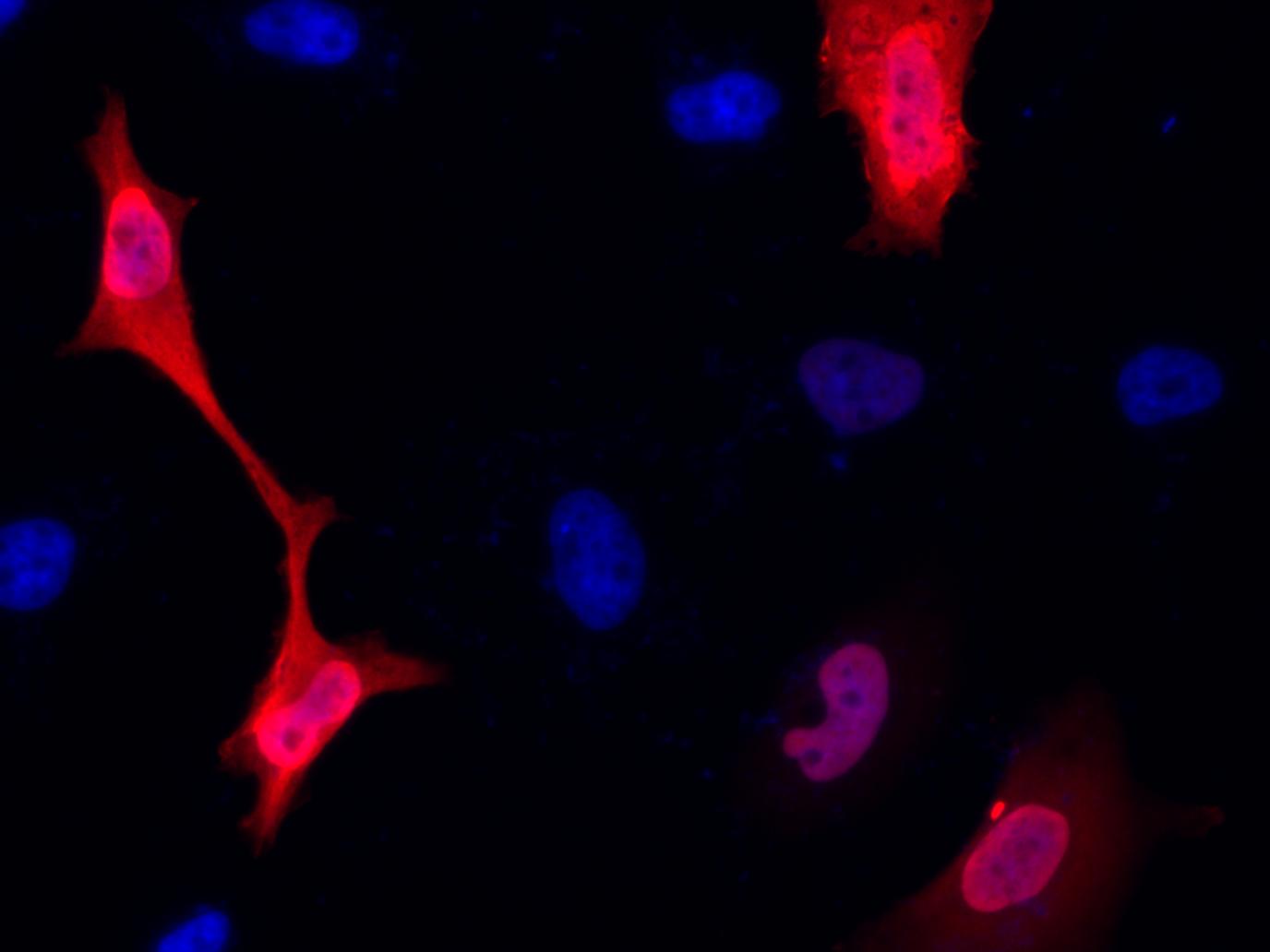

Supplement: S2 File — (ZIP) [file pone.0351194.s002.zip › S2 File/4G.jpg]

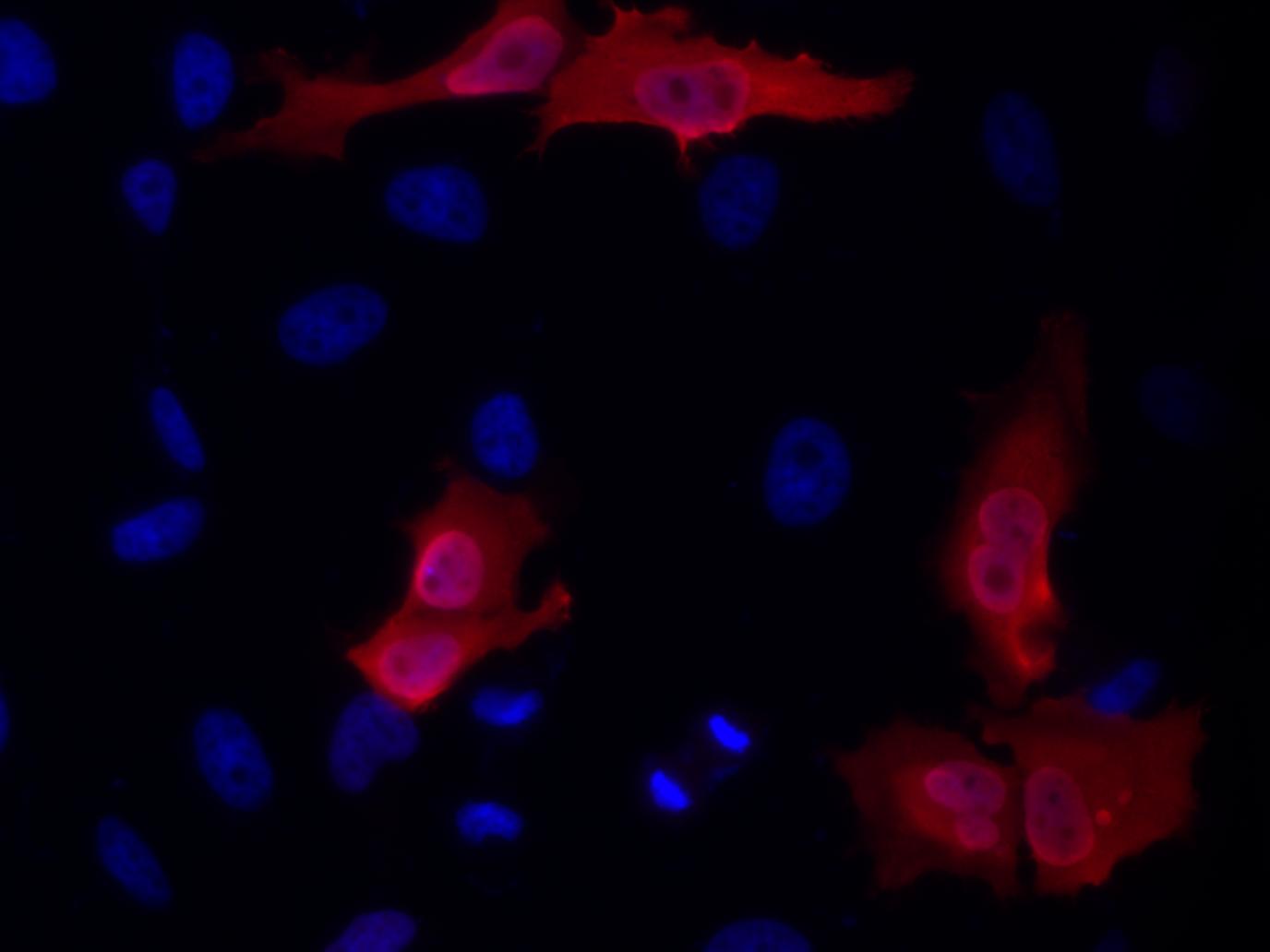

Supplement: S2 File — (ZIP) [file pone.0351194.s002.zip › S2 File/4H.jpg]

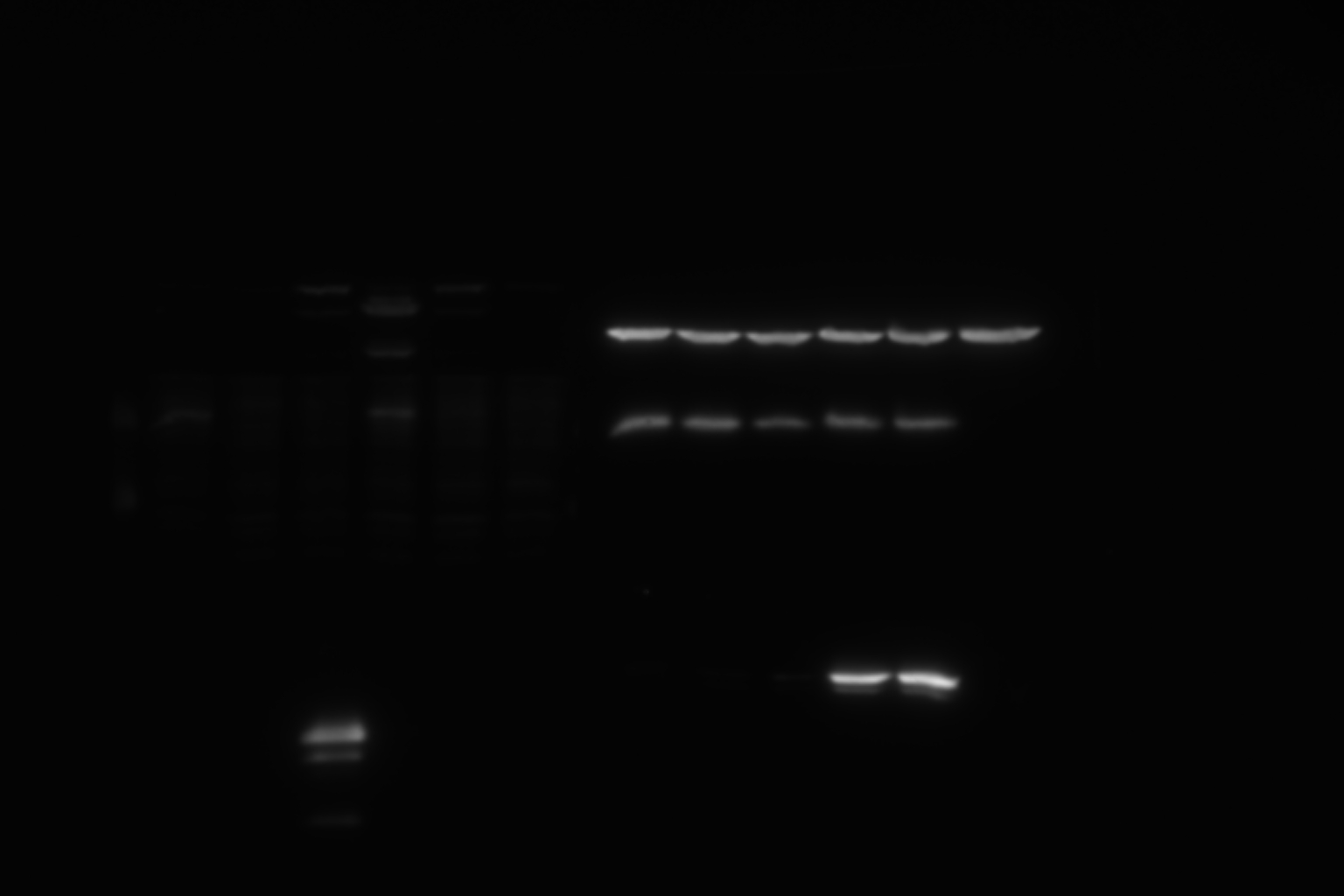

Supplement: S4 File — (ZIP) [file pone.0351194.s004.zip › S4 File/Fig3A/Fig3A_Ulli_D_ING5_20s Kopie.jpg]

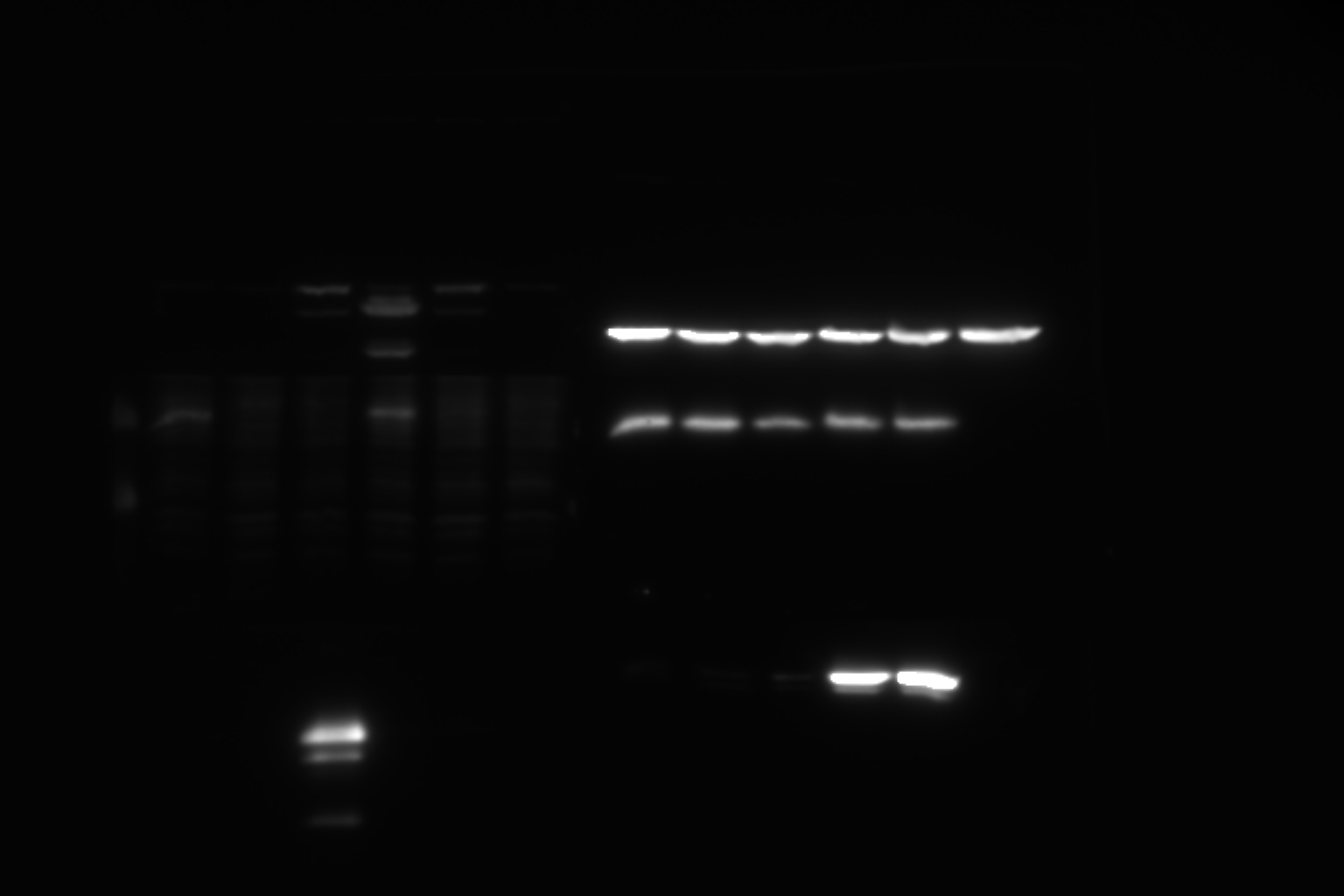

Supplement: S4 File — (ZIP) [file pone.0351194.s004.zip › S4 File/Fig3A/Fig3A_Ulli_D_ING5_40s Kopie.jpg]

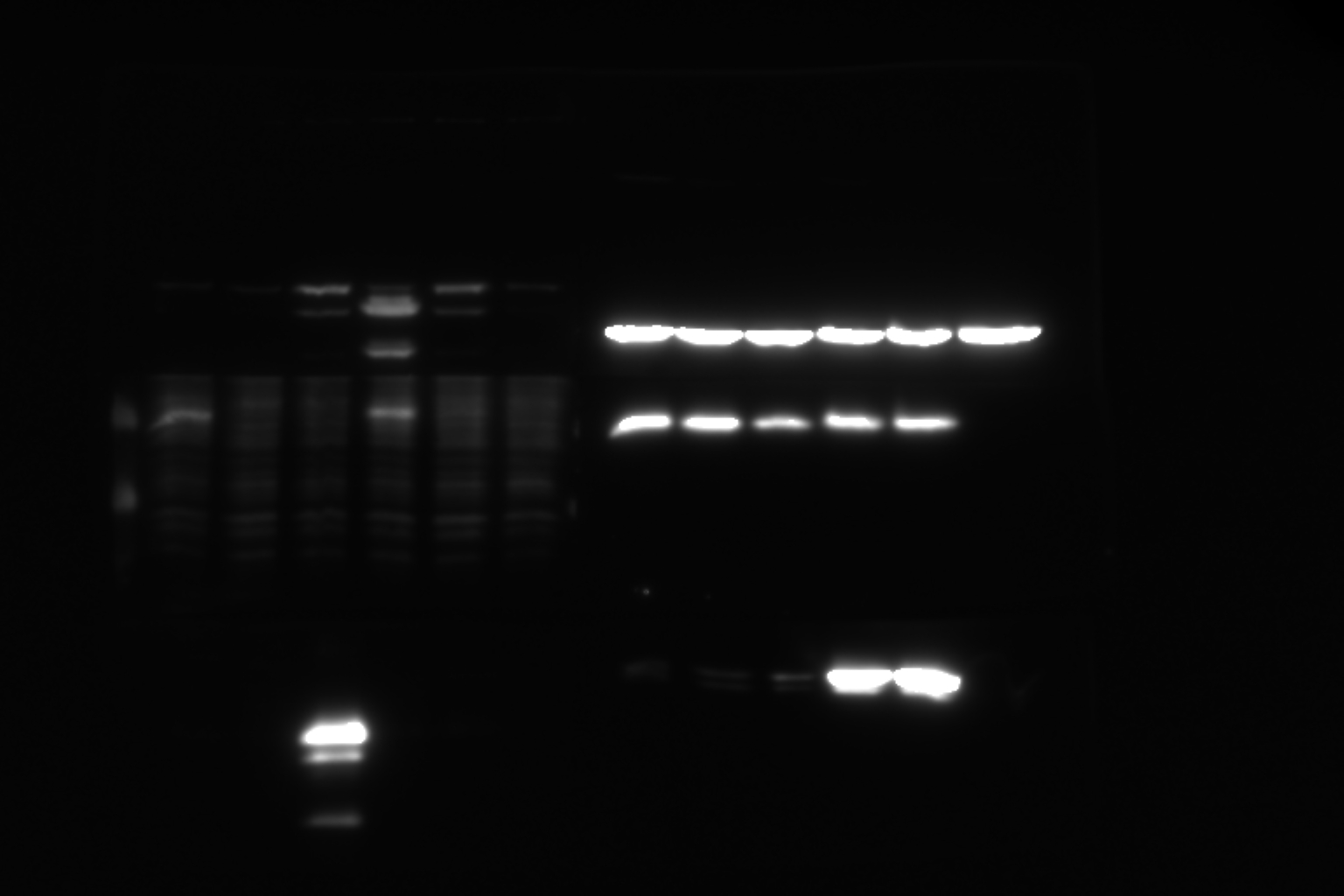

Supplement: S4 File — (ZIP) [file pone.0351194.s004.zip › S4 File/Fig3A/Fig3A_Ulli_D_ING5_80s Kopie.jpg]

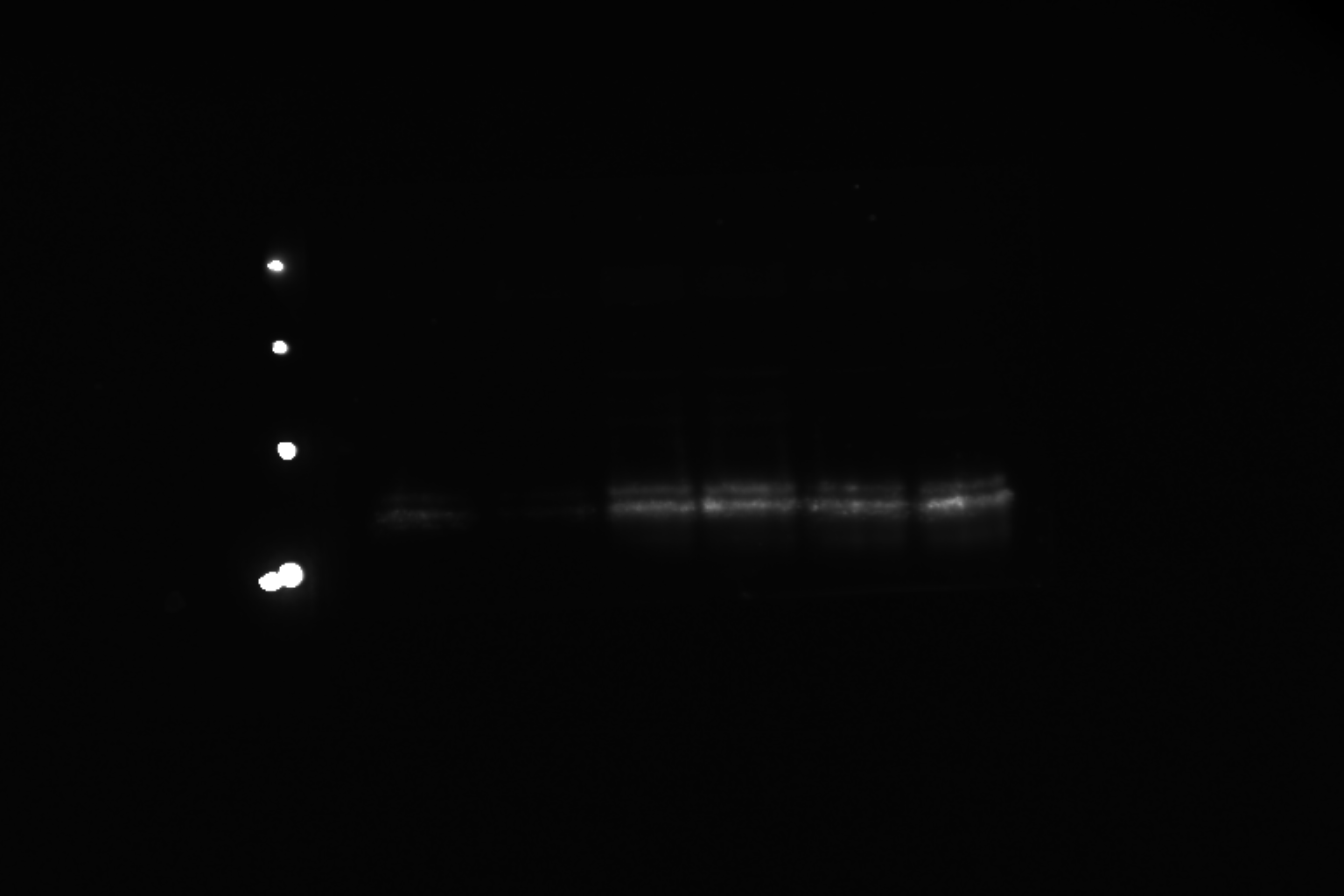

Supplement: S4 File — (ZIP) [file pone.0351194.s004.zip › S4 File/Fig3D/Fig3D_P-ING5_20150219_IP_ING5_P_T152_30s.tif]

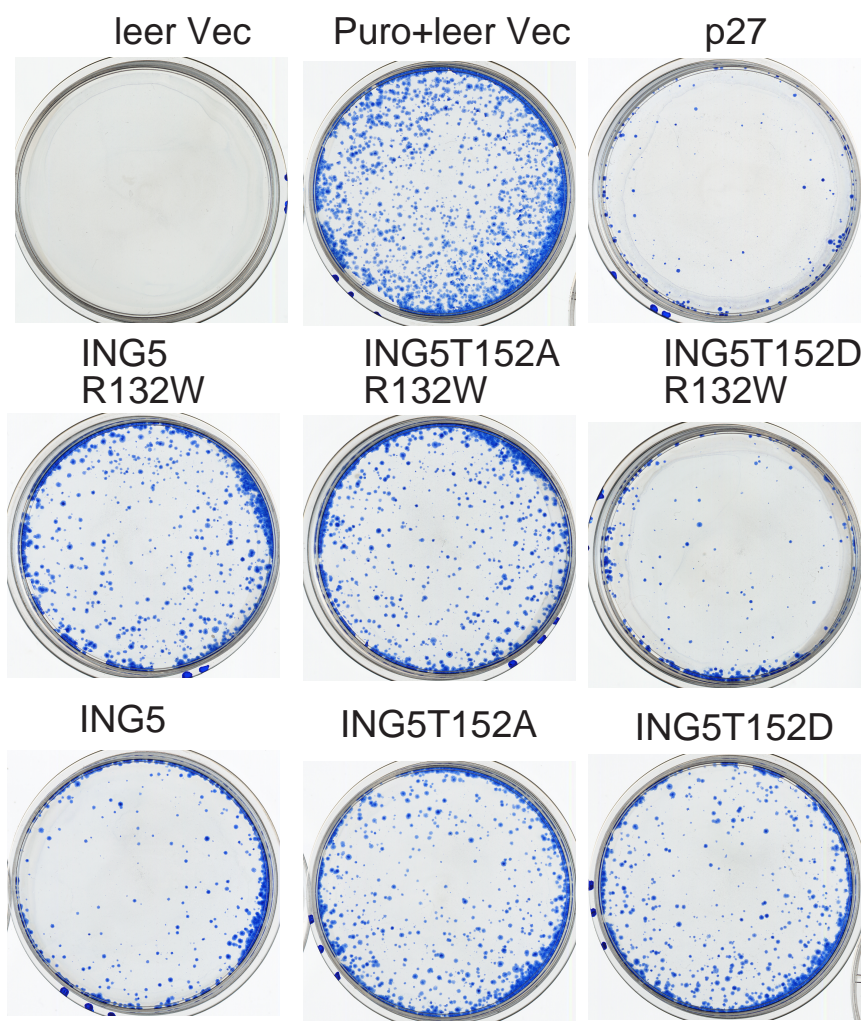

HCT116wt

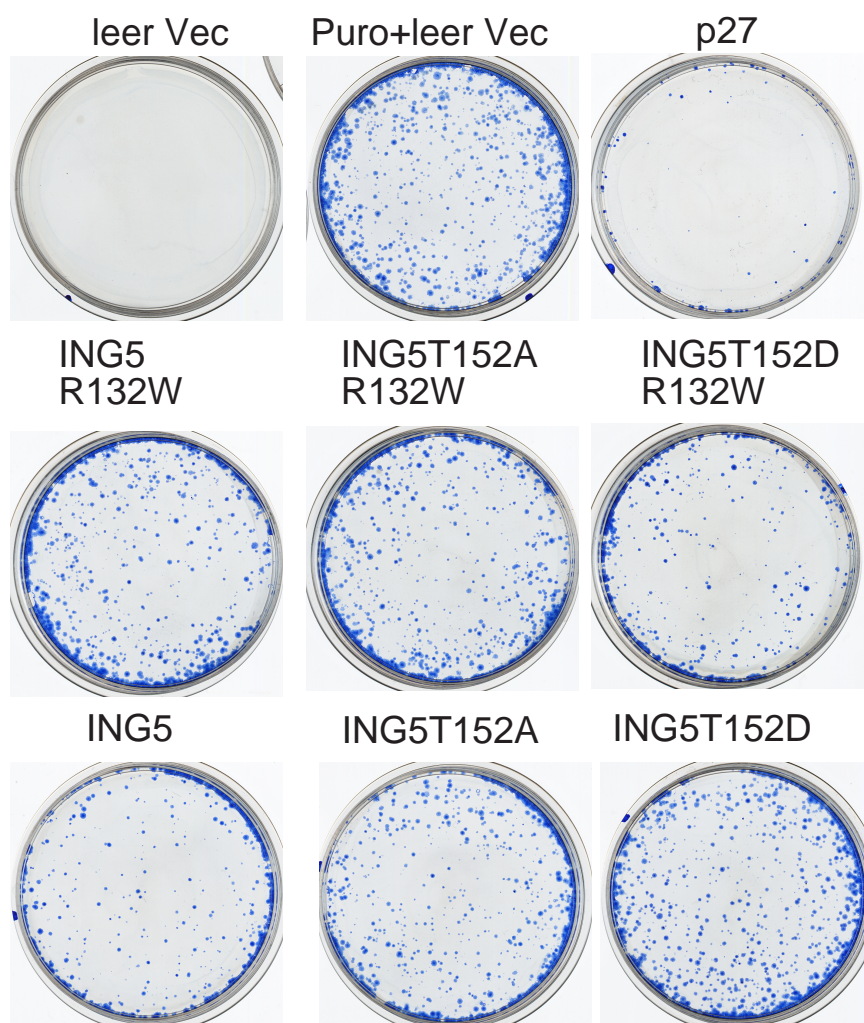

HCT116  
p53 minus

Supplement: S5 File — (ZIP) [file pone.0351194.s005.zip › Fig5A/Ulli_D_75_CFA Kopie.pdf]

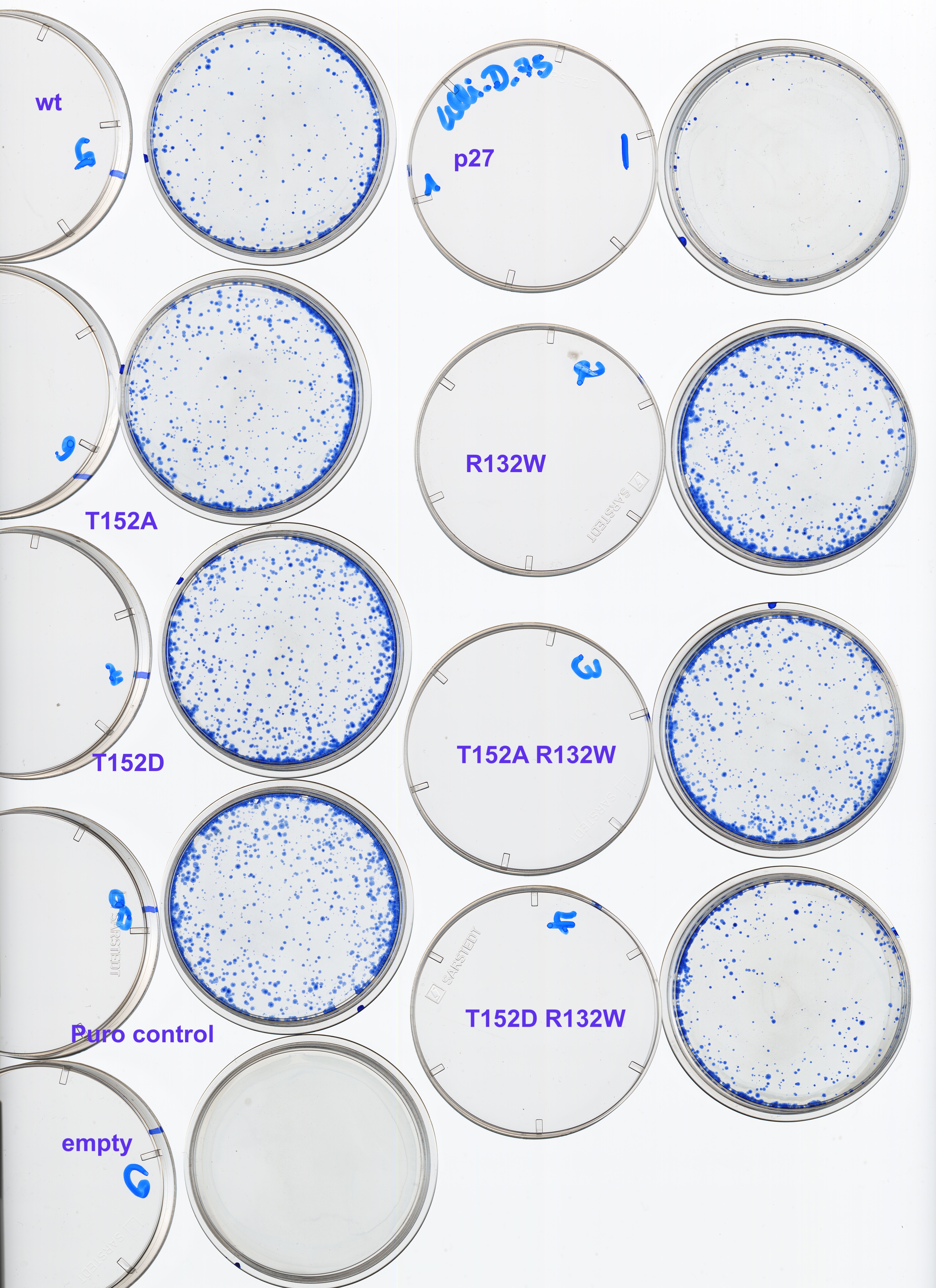

Supplement: S6 File — (ZIP) [file pone.0351194.s006.zip › Fig5B/Ulli_D_75_p53minus Kopie.jpg]

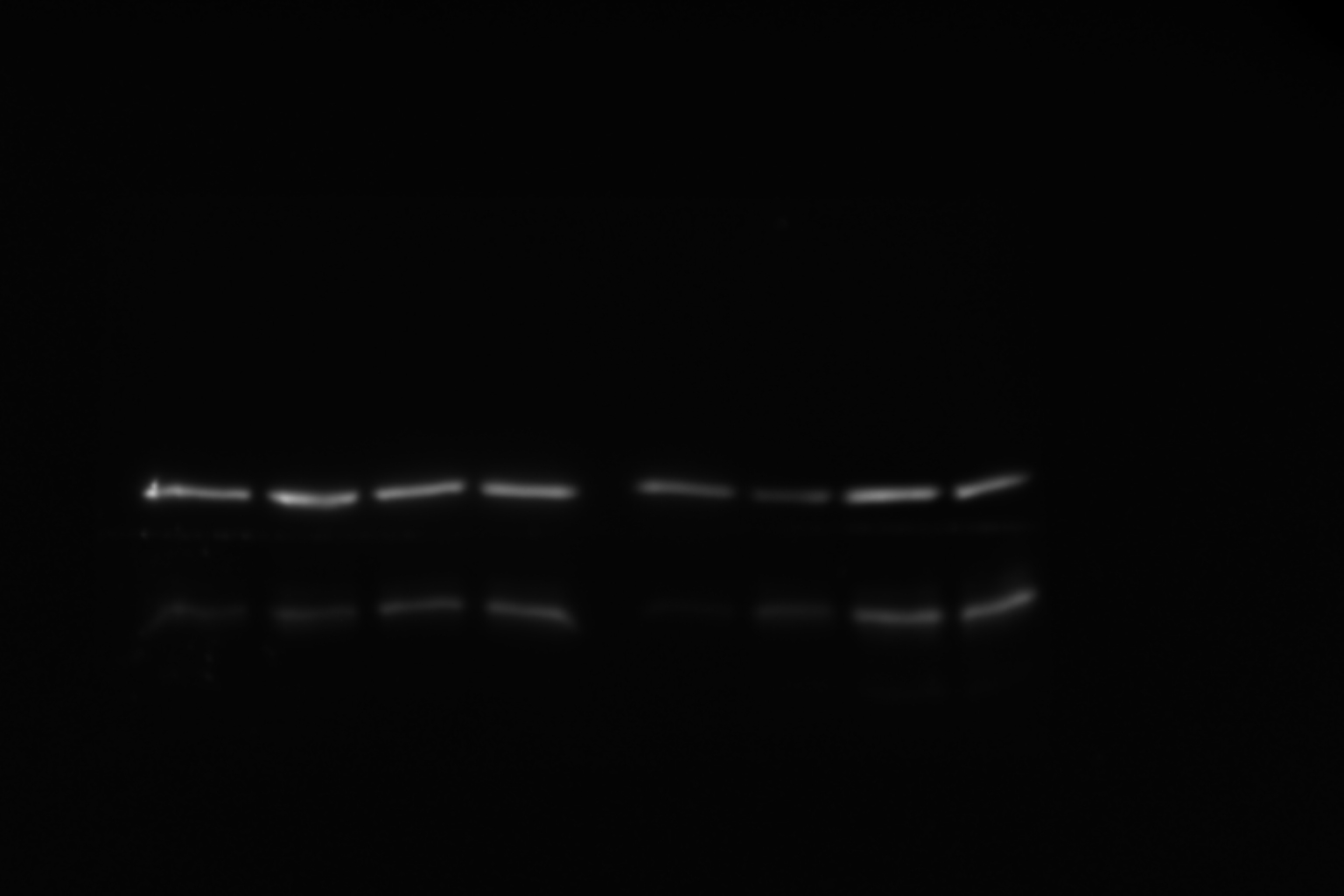

Supplement: S8 File — (ZIP) [file pone.0351194.s008.zip › S8 File/Fig 6A/Fig 6A_ING5_actin 2m Kopie.jpg]

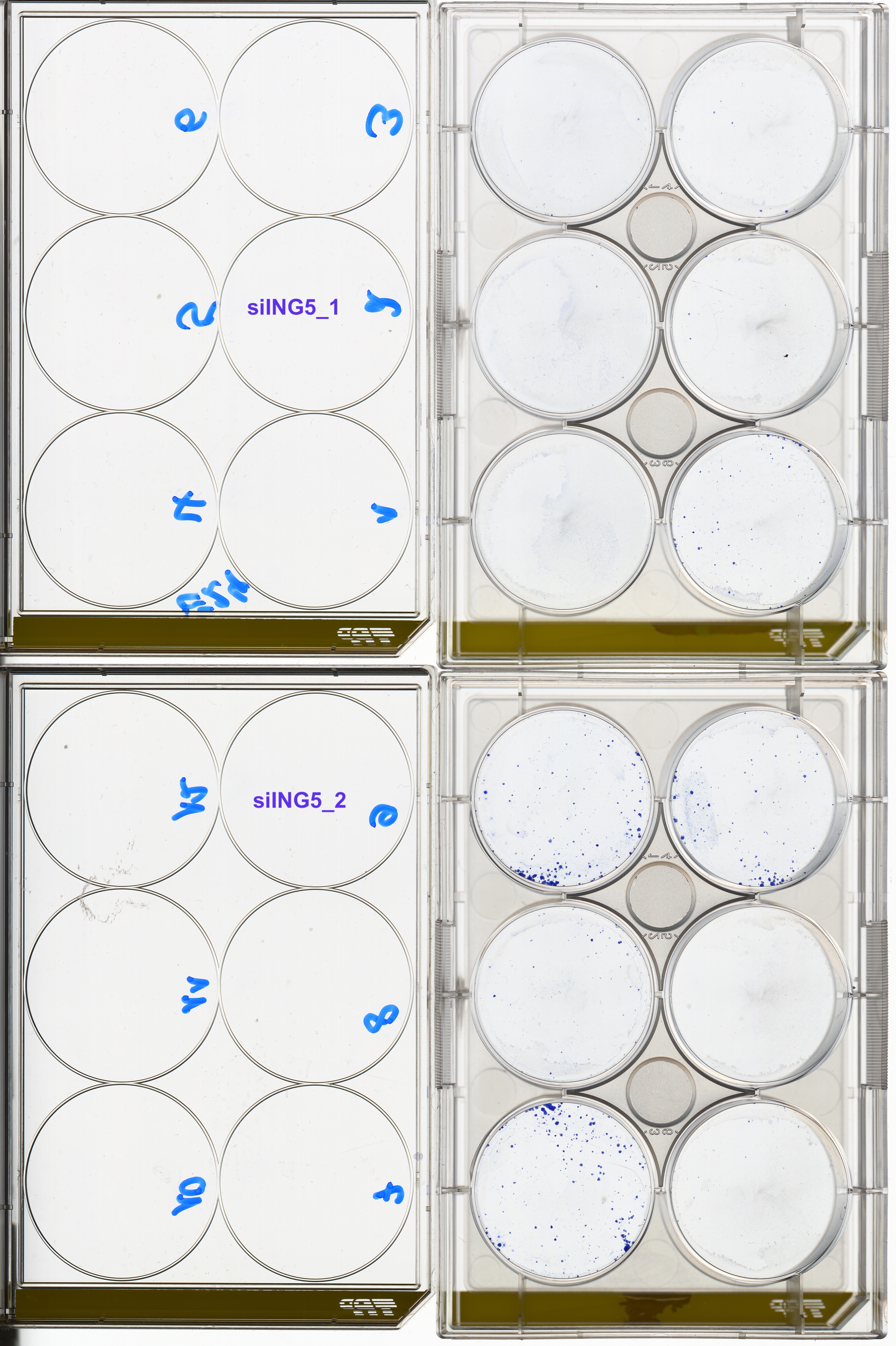

Supplement: S8 File — (ZIP) [file pone.0351194.s008.zip › S8 File/Fig 6B/Ulli_E_24_1.jpg]

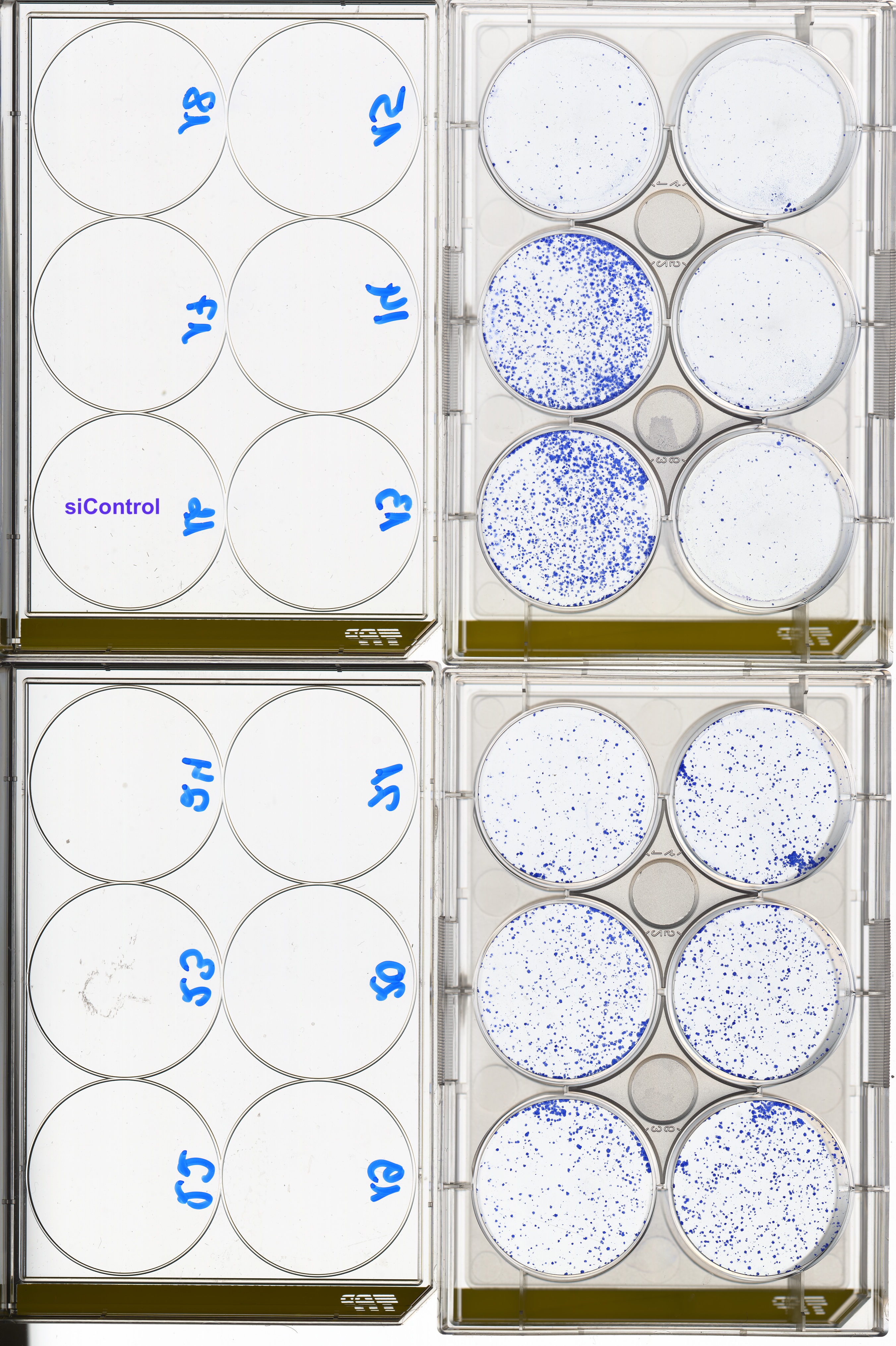

Supplement: S8 File — (ZIP) [file pone.0351194.s008.zip › S8 File/Fig 6B/Ulli_E_24_2.jpg]

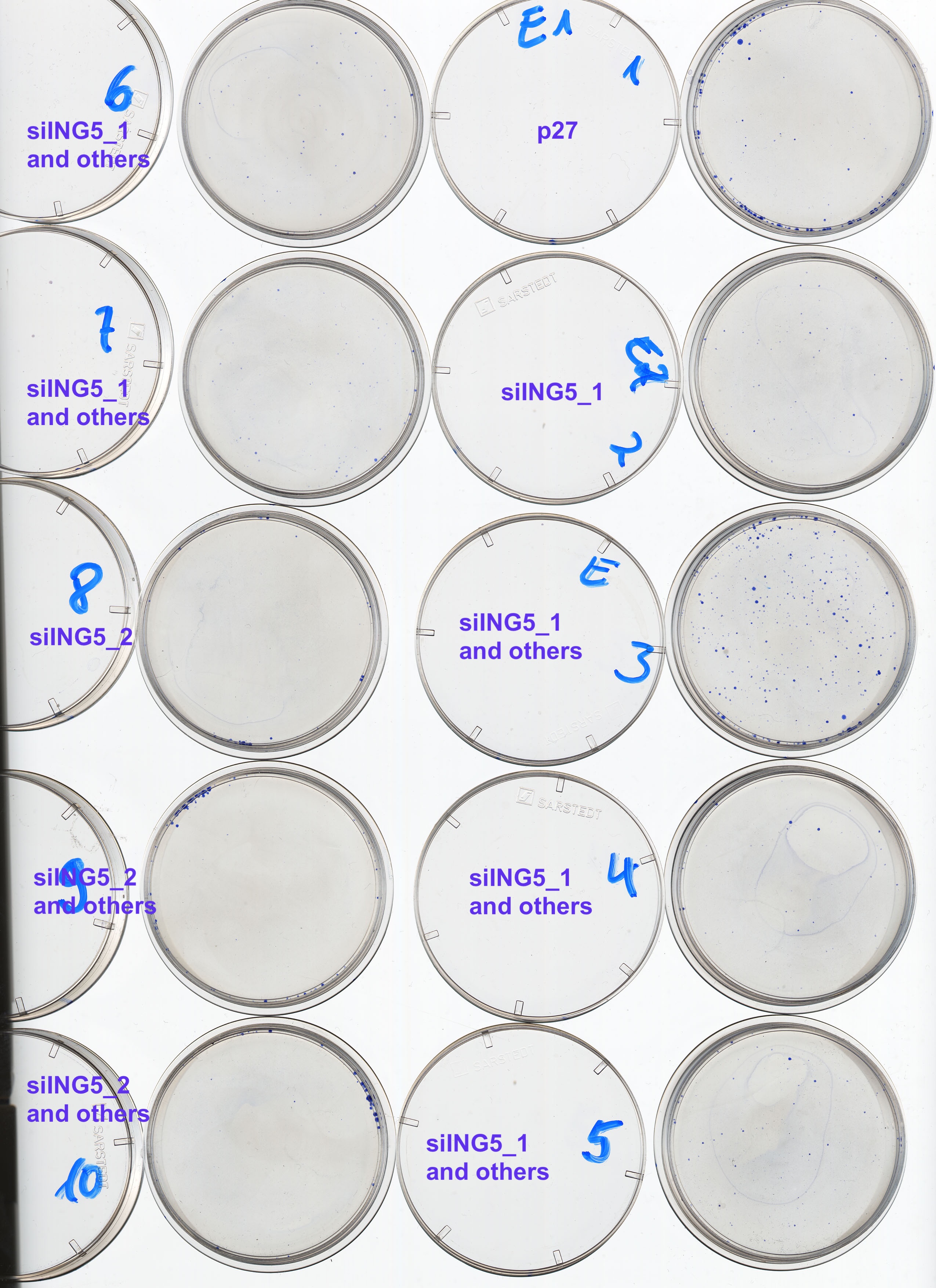

Supplement: S8 File — (ZIP) [file pone.0351194.s008.zip › S8 File/Fig 6C/Ulli_E_1_1.jpg]

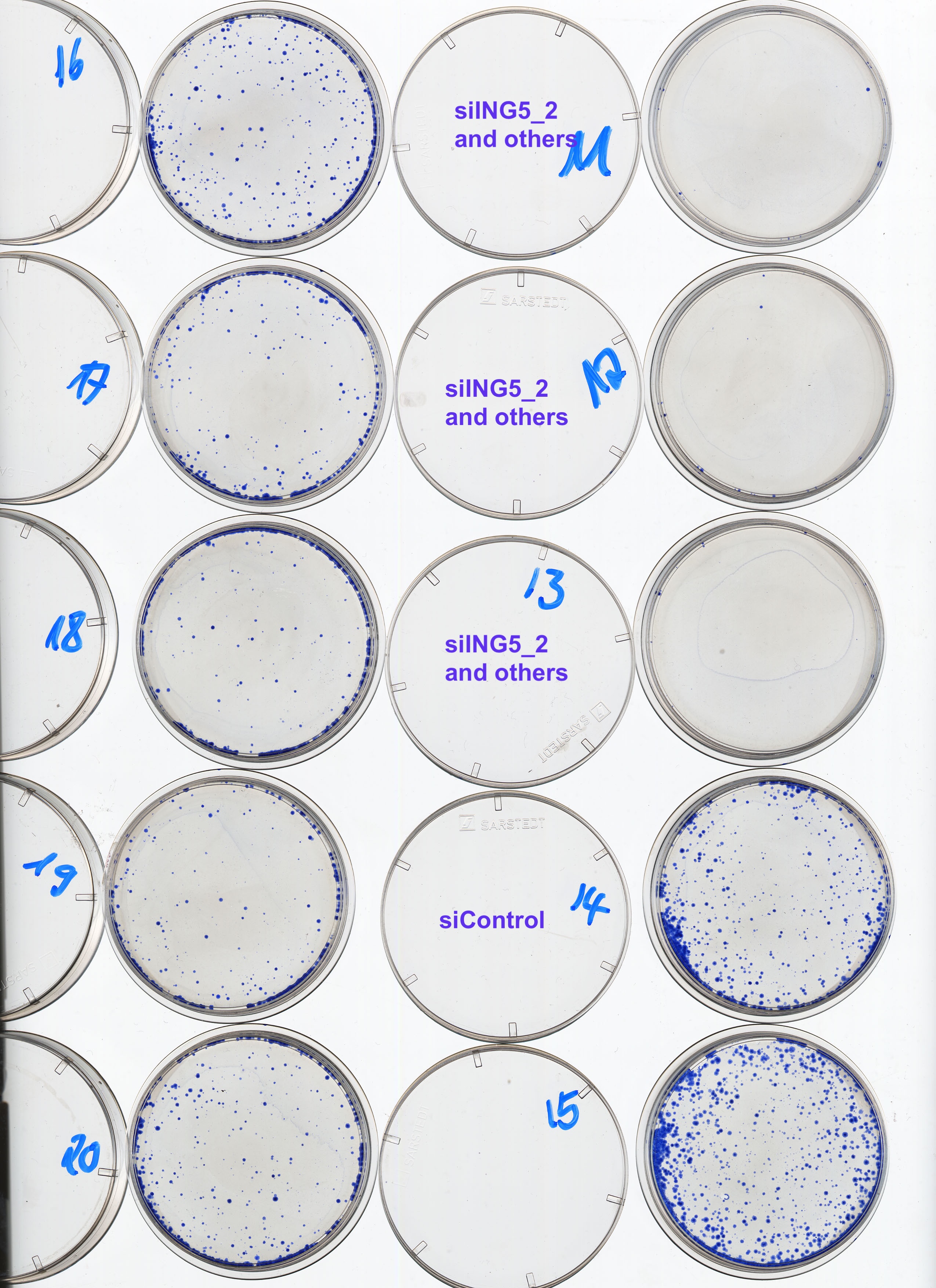

Supplement: S8 File — (ZIP) [file pone.0351194.s008.zip › S8 File/Fig 6C/Ulli_E_1_2.jpg]

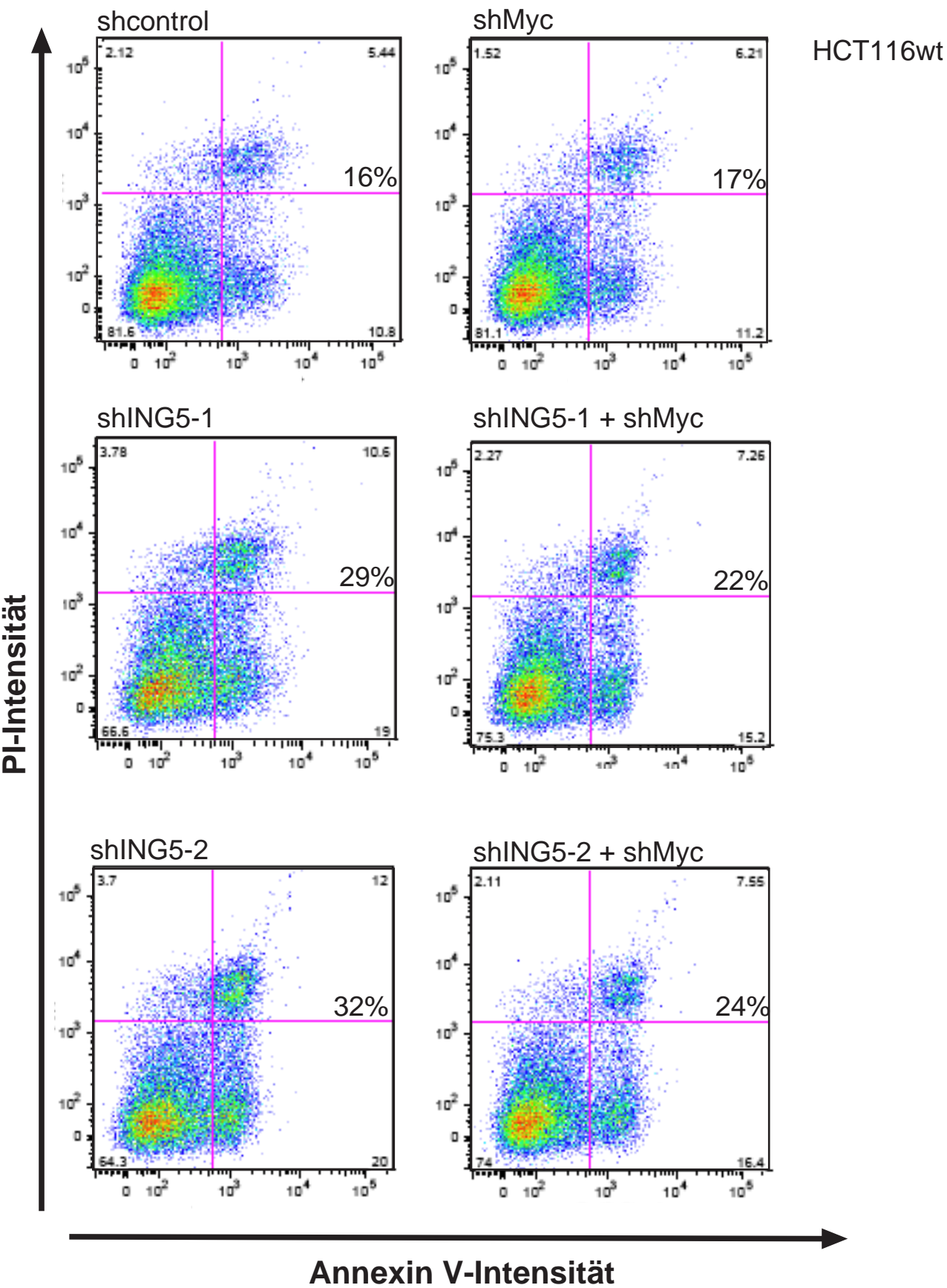

Supplement: S9 File — (ZIP) [file pone.0351194.s009.zip › S9 File/Fig 7A/Annexin_shING5_shMycp53 Kopie.pdf]
