# Supplementary material for: Expression of Concern: ING5 is phosphorylated by CDK2 and controls cell proliferation independently of p53
Source: PLoS One. 2026 Jun 9;21(6):e0351194. doi: 10.1371/journal.pone.0351194 (PMC13249149; doi:10.1371/journal.pone.0351194)
Supplement: S7 File — (ZIP) [file pone.0351194.s007.zip › Fig5F&G/271109 cell cycle Kopie/D_77_cellcyleING5mut.pdf]

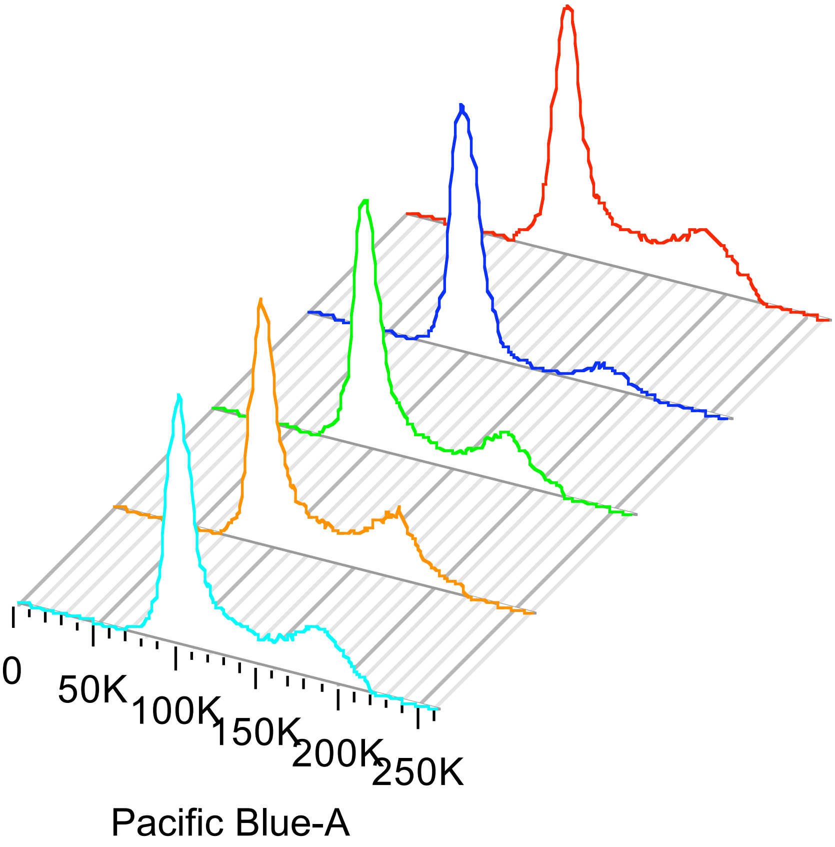

Pacific Blue-A, SSC-A subset

	HCT116_7 wt VB.fcs	100
	HCT116_6 wt VB.fcs	100
	HCT116_5 wt VB.fcs	100
	HCT116_1 wt VB.fcs	100
	HCT116_8 GFP wt VB.fcs	100

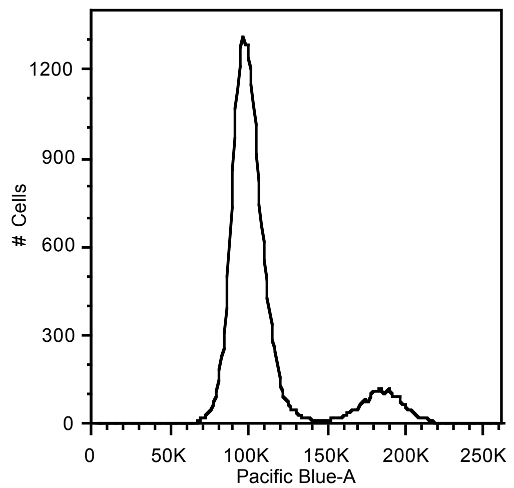

Pacific Blue-A, SSC-A subset
HCT116_1 wt VB.fcs
Event Count: 33946

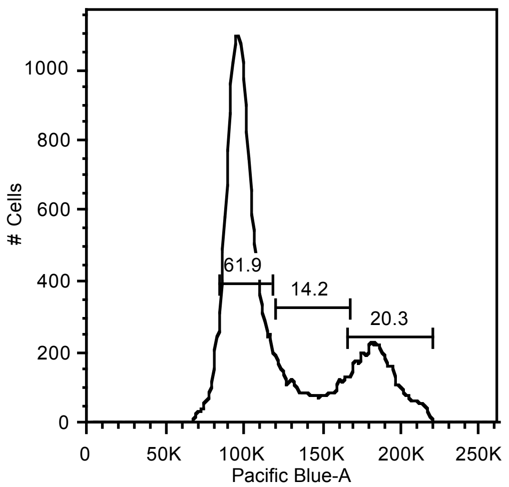

Pacific Blue-A, SSC-A subset
HCT116_5 wt VB.fcs
Event Count: 35579

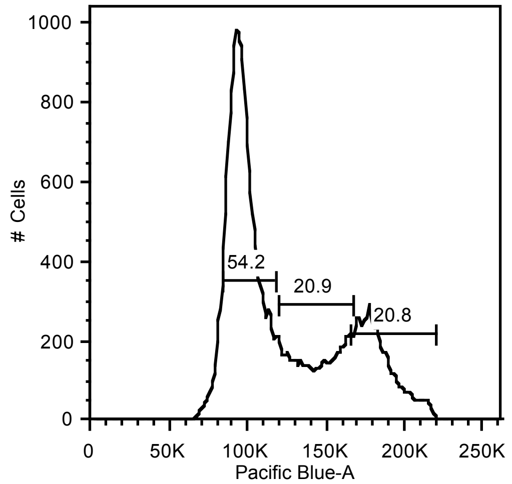

Pacific Blue-A, SSC-A subset
HCT116_6 wt VB.fcs
Event Count: 35769

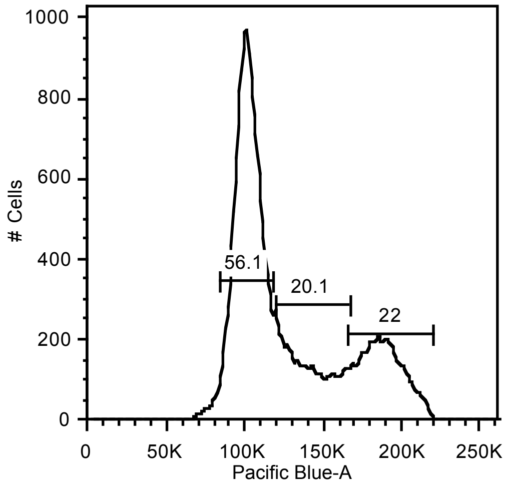

Pacific Blue-A, SSC-A subset
HCT116_7 wt VB.fcs
Event Count: 33511

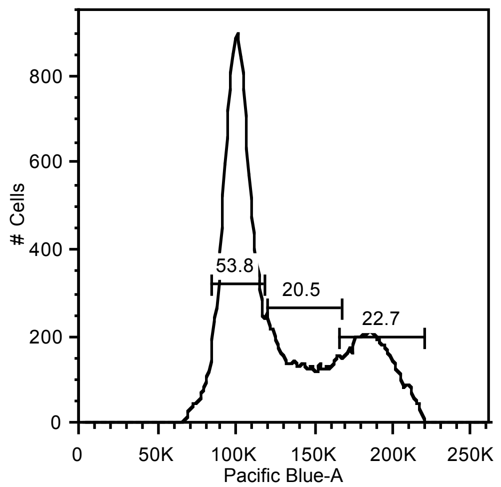

Pacific Blue-A, SSC-A subset
HCT116_8 GFP wt VB.fcs
Event Count: 34732

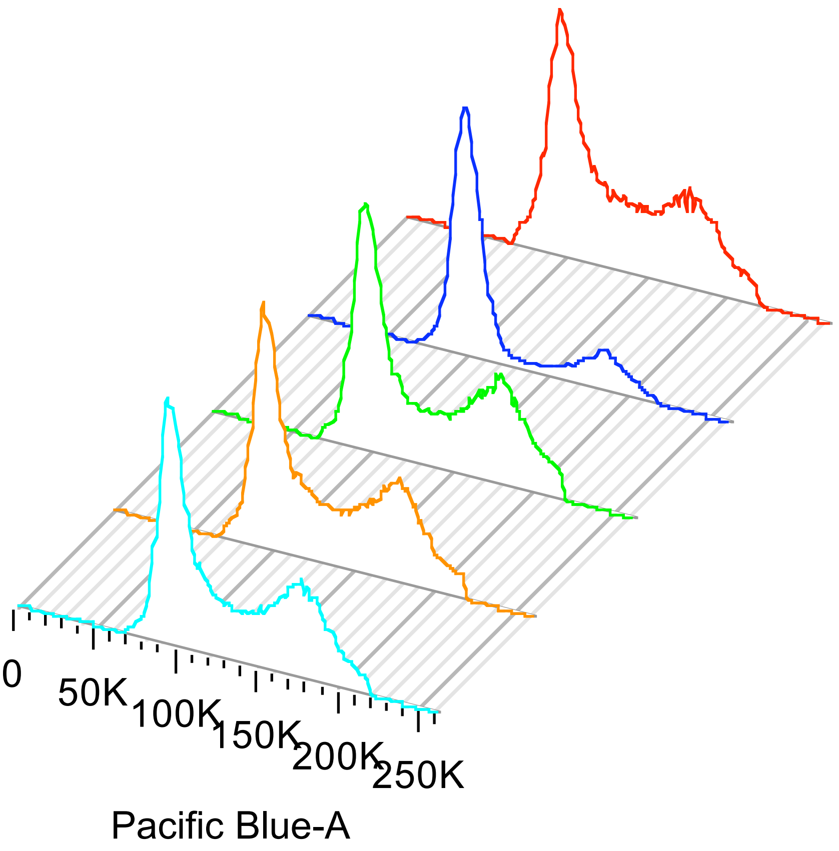

Pacific Blue-A, SSC-A subset

HCT116_7 minus VB.fcs	100
HCT116_6 minus VB.fcs	100
HCT116_5 mut minus VB.fcs	100
HCT116_1 minus wt.fcs	100
HCT116_8 minus VB.fcs	100

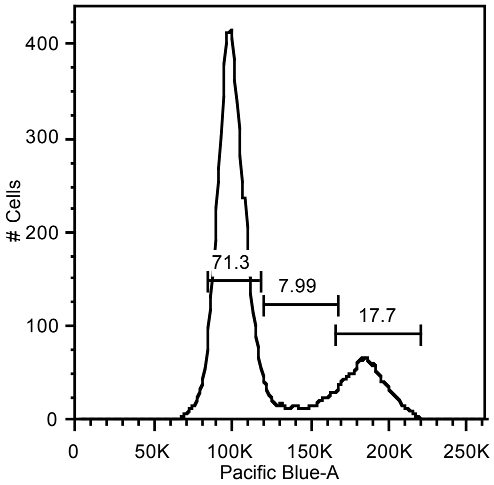

Pacific Blue-A, SSC-A subset
HCT116_1 minus wt.fcs
Event Count: 11522

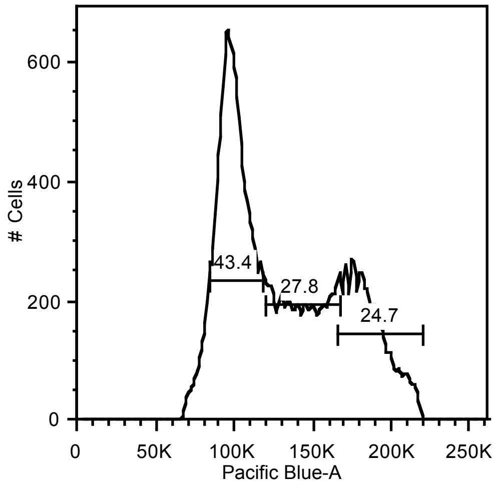

Pacific Blue-A, SSC-A subset
HCT116_8 minus VB.fcs
Event Count: 33861

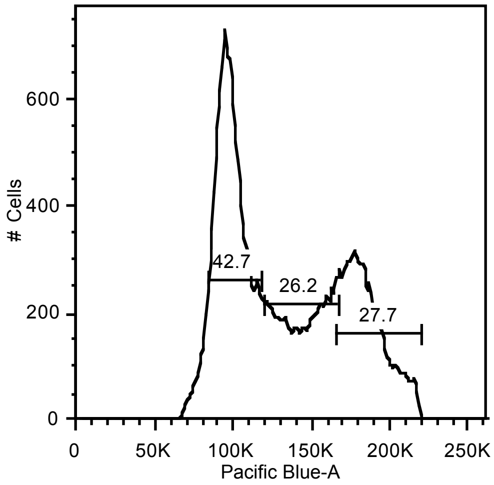

Pacific Blue-A, SSC-A subset
HCT116_6 minus VB.fcs
Event Count: 35708

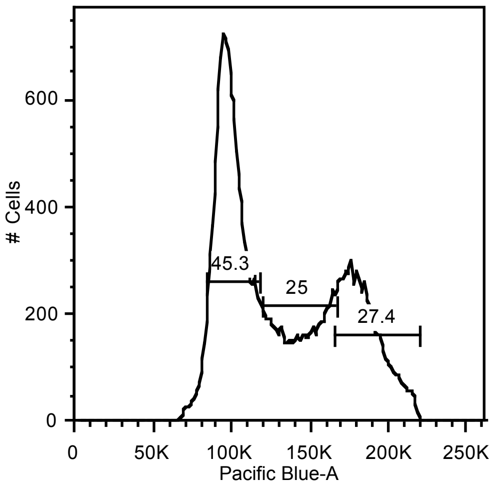

Pacific Blue-A, SSC-A subset
HCT116_7 minus VB.fcs
Event Count: 33572

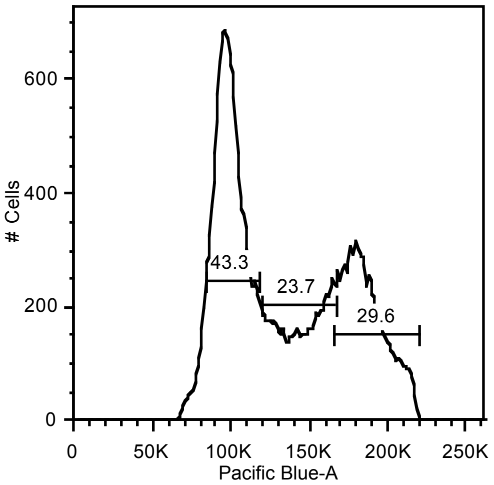

Pacific Blue-A, SSC-A subset
HCT116_5 mut minus VB.fcs
Event Count: 34849
