# Supplementary material for: Expression of Concern: ING5 is phosphorylated by CDK2 and controls cell proliferation independently of p53
Source: PLoS One. 2026 Jun 9;21(6):e0351194. doi: 10.1371/journal.pone.0351194 (PMC13249149; doi:10.1371/journal.pone.0351194)
Supplement: S8 File — (ZIP) [file pone.0351194.s008.zip › S8 File/Fig6rev_20260123.pdf]

Linzen et al. Figure 6 revised

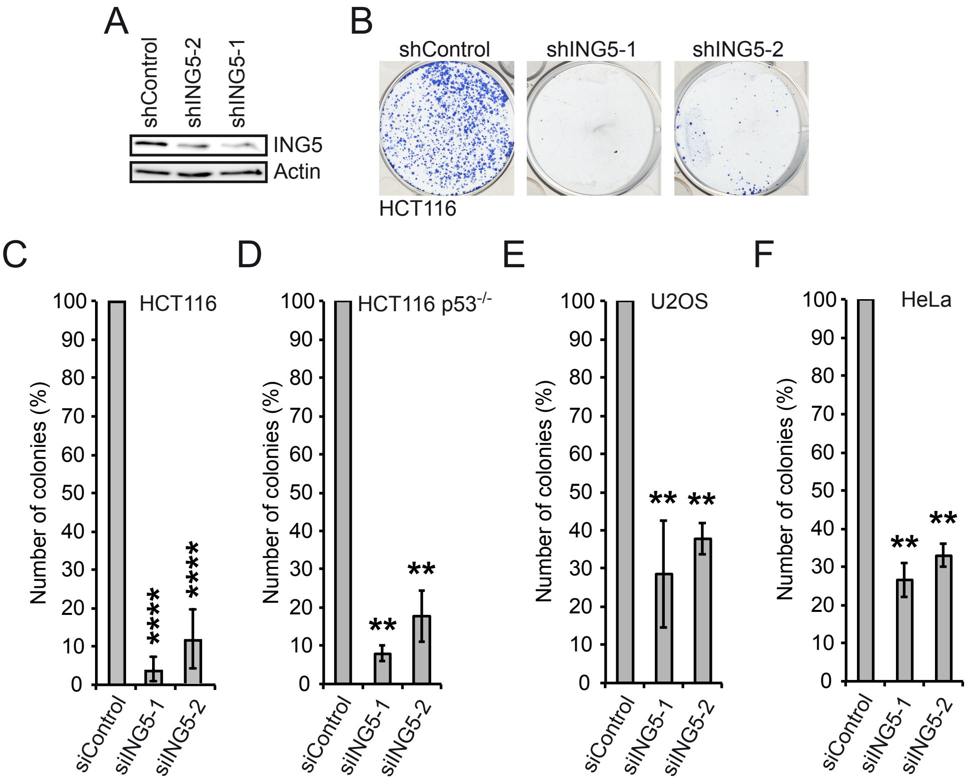

Fig 6. Knockdown of ING5 inhibits cell proliferation in tumor cells independent of p53.
(A) HEK293 cells were transfected with the indicated pSuper constructs. The expression of ING5 was analyzed using mAb 7A11. Actin is shown for control.
(B) HCT116 cells were co-transfected with plasmids expressing the indicated shRNAs and a puromycin resistance plasmid. The puromycin selected cells were evaluated 10 days after transfection by staining with methylene blue.
(C-F) Quantification of 2-4 independent experiments with HCT116 (C), HCT116-p53-/- (D), U2OS (E) and HeLa (F) cells. ** p<0.01, ****p<0.0001.
For panel C, ordinary one-way ANOVA / multiple comparison of four independent experiments were used.
