# Supplementary material for: Expression of Concern: ING5 is phosphorylated by CDK2 and controls cell proliferation independently of p53
Source: PLoS One. 2026 Jun 9;21(6):e0351194. doi: 10.1371/journal.pone.0351194 (PMC13249149; doi:10.1371/journal.pone.0351194)
Supplement: S9 File — (ZIP) [file pone.0351194.s009.zip › S9 File/Fig 7A/Ulli_E_33_Annexin PI shING5 Kopie/Auswertung.jo - Layout Editor.pdf]

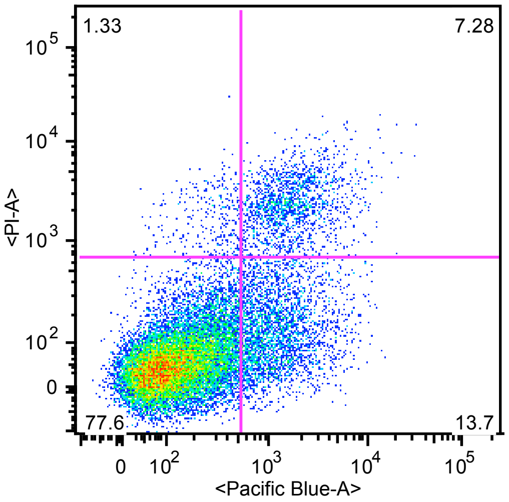

transfected cells
HCT116_shControl.fcs
Event Count: 22336

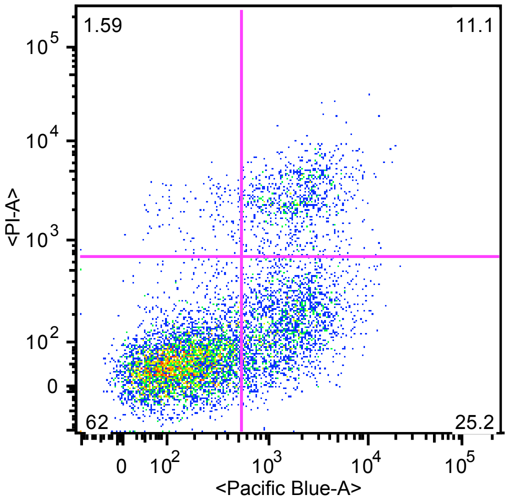

transfected cells
HCT116_shING5_1.fcs
Event Count: 7859

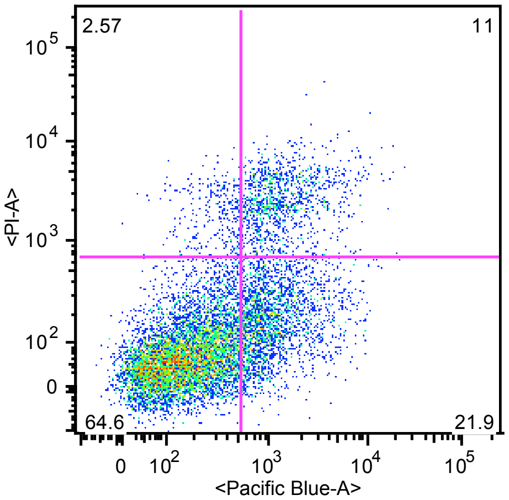

transfected cells
HCT116_shING5_1 DMSO.fcs
Event Count: 10985

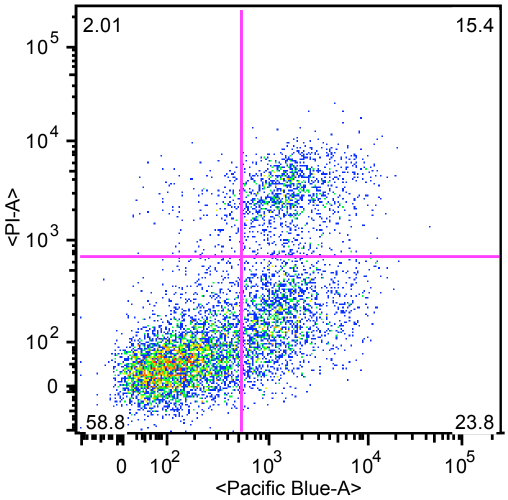

transfected cells
HCT116_shING5_1 resING5_1.fcs
Event Count: 8395

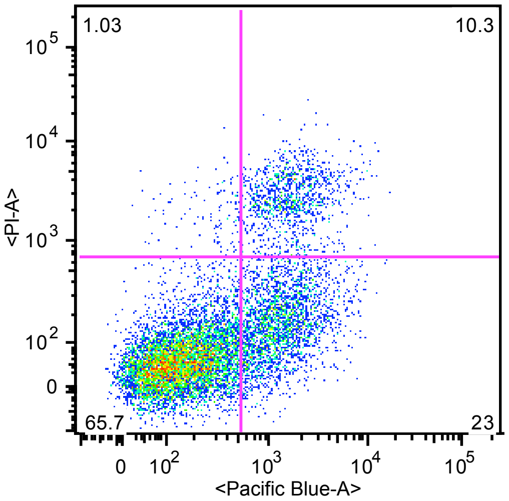

transfected cells
HCT116_shING5_1 Bcl2.fcs
Event Count: 11309

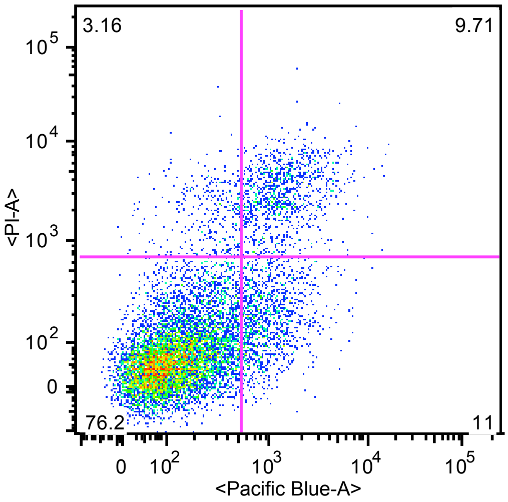

transfected cells
HCT116_shING5_1 Z-VAD.fcs
Event Count: 10802

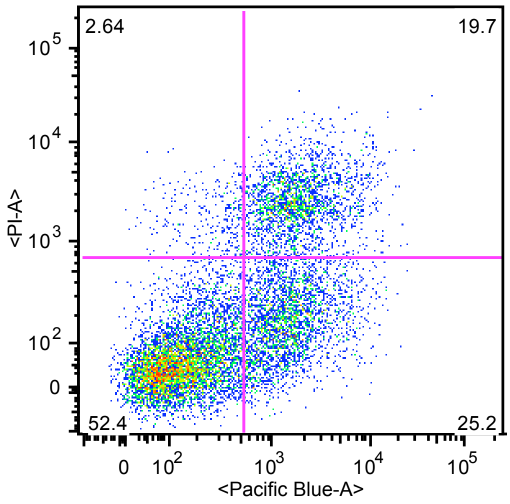

transfected cells
HCT116_shING5_2.fcs
Event Count: 11022

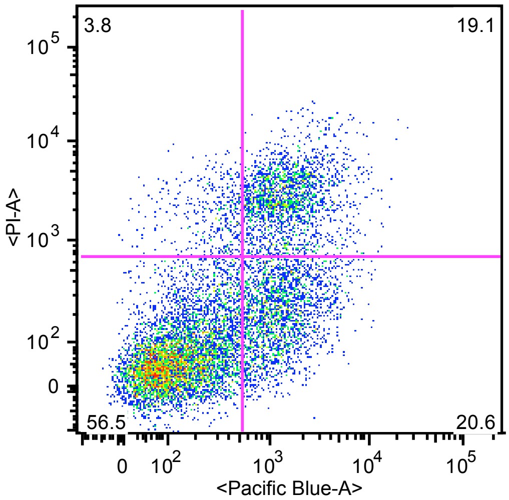

transfected cells
HCT116_shING5_2 DMSO.fcs
Event Count: 10801

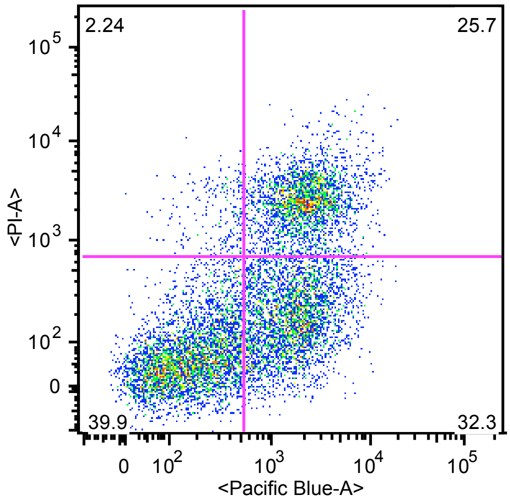

transfected cells
HCT116_shING5_2 resING5_2.fcs
Event Count: 10791

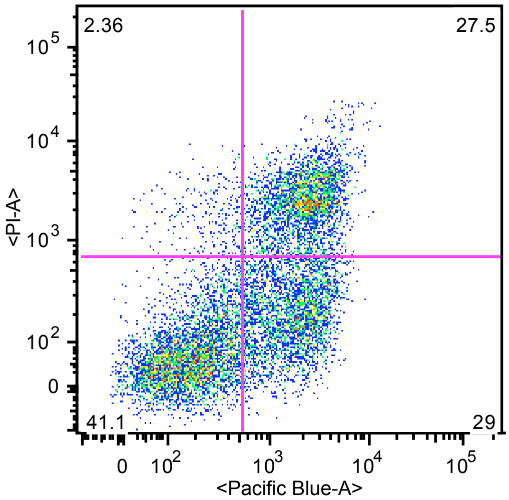

transfected cells
HCT116_shING5_2 Bcl2.fcs
Event Count: 10992

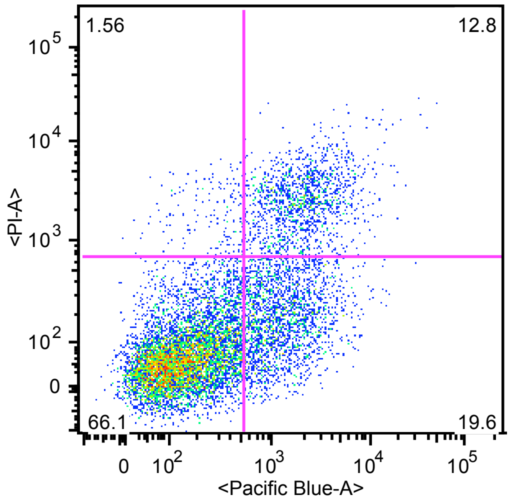

transfected cells
HCT116_shING5_2 Z-VAD.fcs
Event Count: 10404
