# Supplementary material for: Expression of Concern: ING5 is phosphorylated by CDK2 and controls cell proliferation independently of p53
Source: PLoS One. 2026 Jun 9;21(6):e0351194. doi: 10.1371/journal.pone.0351194 (PMC13249149; doi:10.1371/journal.pone.0351194)
Supplement: S9 File — (ZIP) [file pone.0351194.s009.zip › S9 File/Fig 7A/Ulli_E_55 Kopie/080910_p53minus.pdf]

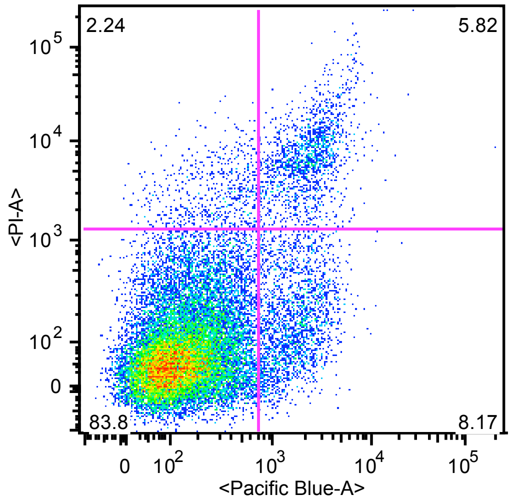

tranzifierte Zellen
HCT116 p53 minus_shControl.fcs
Event Count: 26203

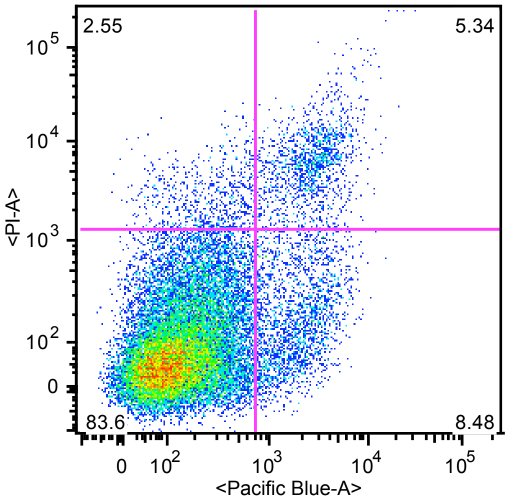

tranzifierte Zellen
HCT116 p53 minus_shMyc.fcs
Event Count: 26387

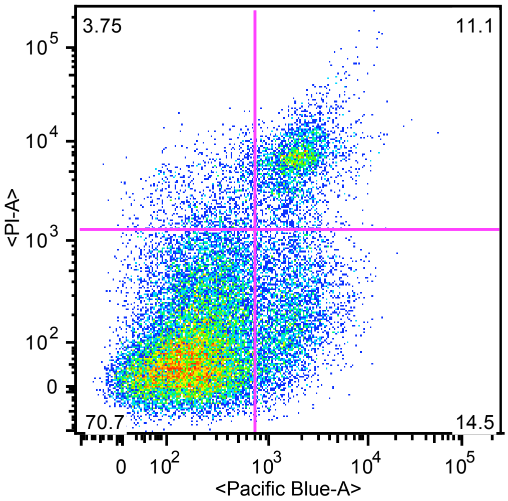

tranzifierte Zellen
HCT116 p53 minus_shING5_1.fcs
Event Count: 27681

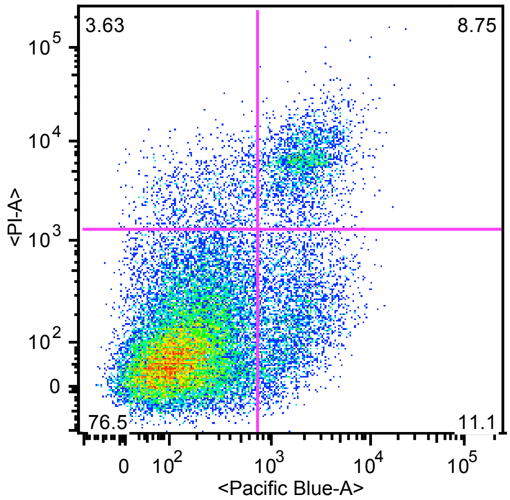

tranzifierte Zellen
HCT116 p53 minus_shING5_1 shMyc.fcs
Event Count: 27577

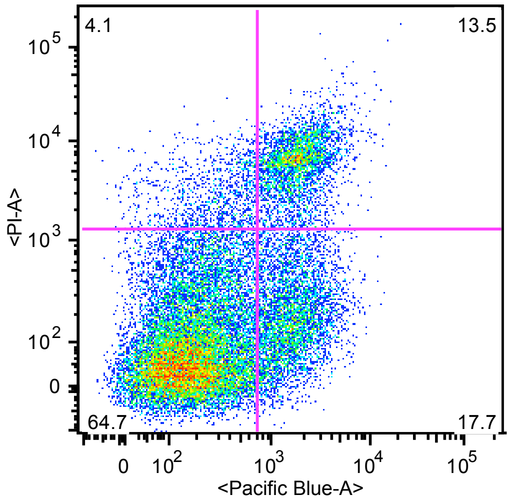

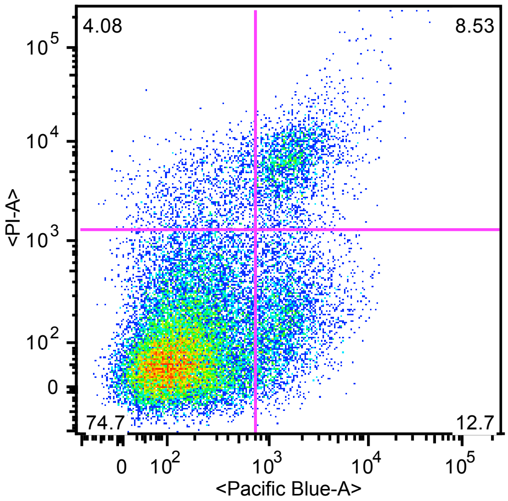

;
