# Supplementary material for: Expression of Concern: ING5 is phosphorylated by CDK2 and controls cell proliferation independently of p53
Source: PLoS One. 2026 Jun 9;21(6):e0351194. doi: 10.1371/journal.pone.0351194 (PMC13249149; doi:10.1371/journal.pone.0351194)
Supplement: S9 File — (ZIP) [file pone.0351194.s009.zip › S9 File/Fig 7A/Ulli_E_55 Kopie/080910_p53wt.pdf]

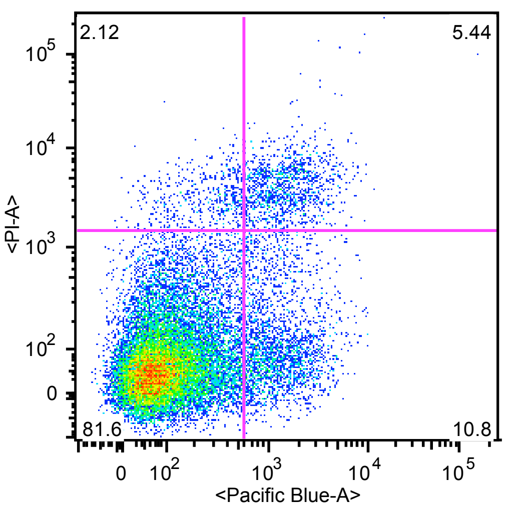

transfected cells
HCT116 wt_shControl.fcs
Event Count: 23059

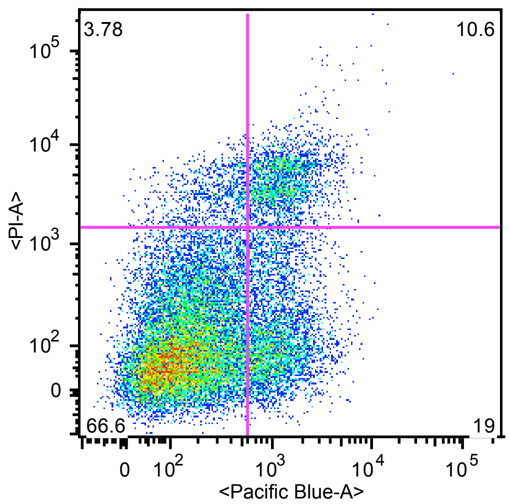

transfected cells
HCT116 wt_shING5_1.fcs
Event Count: 22917

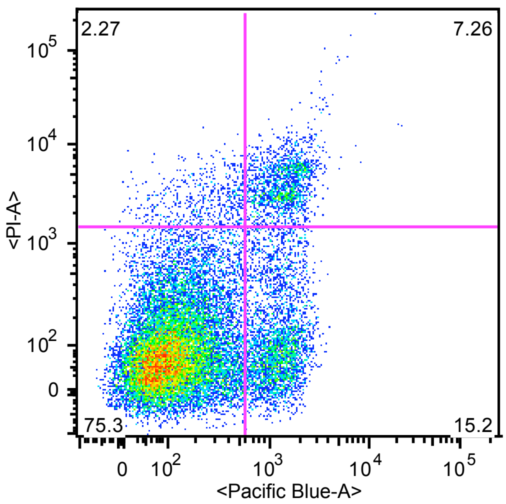

transfected cells
HCT116 wt_shING5_1 shMyc.fcs
Event Count: 23451

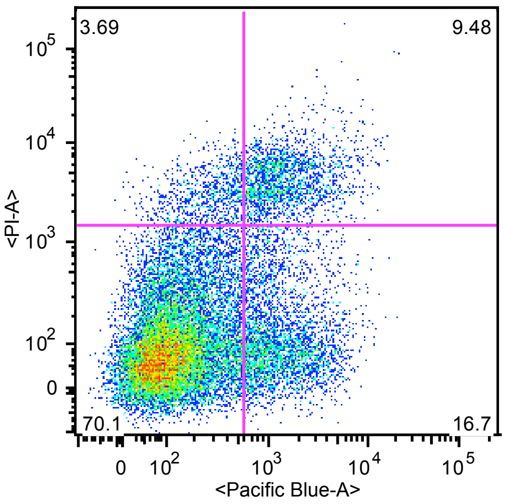

transfected cells
HCT116 wt_shING5_1 Myc.fcs
Event Count: 23142

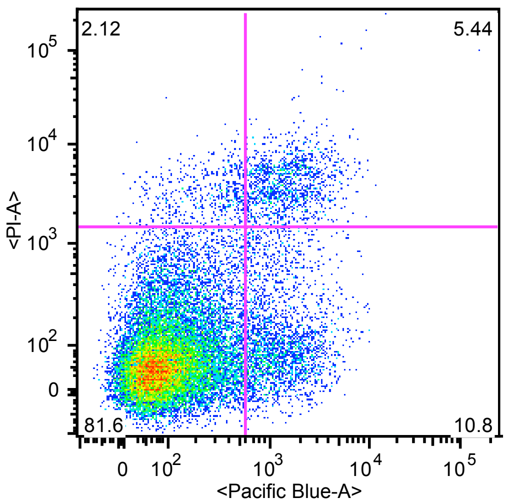

transfected cells
HCT116 wt_shControl.fcs
Event Count: 23059

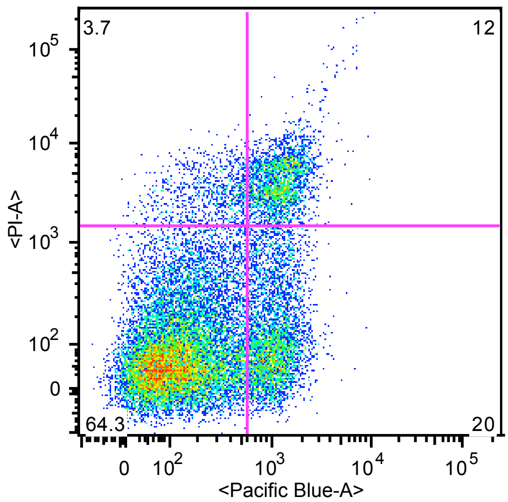

transfected cells
HCT116 wt_shING5_2.fcs
Event Count: 23358

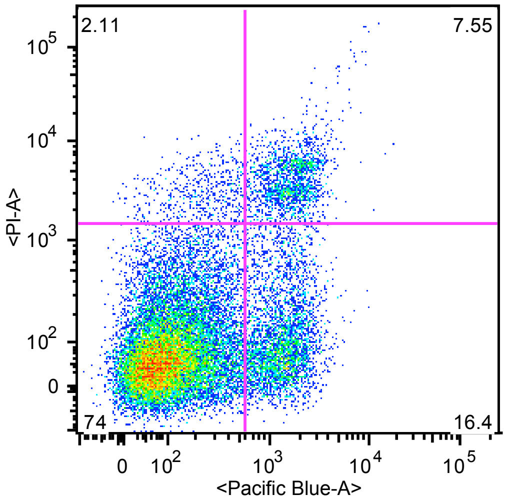

transfected cells
HCT116 wt_shING5_2 shMyc.fcs
Event Count: 22917

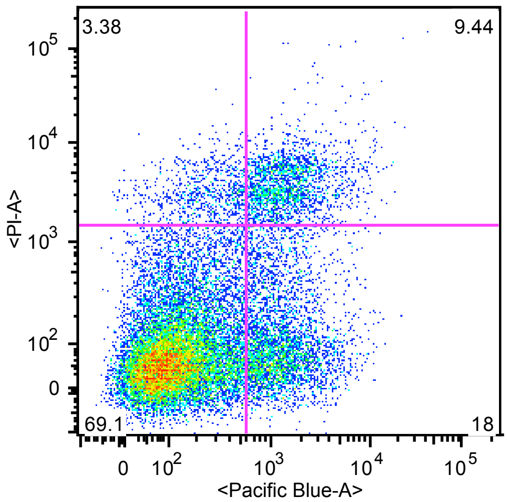

transfected cells
HCT116 wt_shING5_2 Myc.fcs
Event Count: 23115

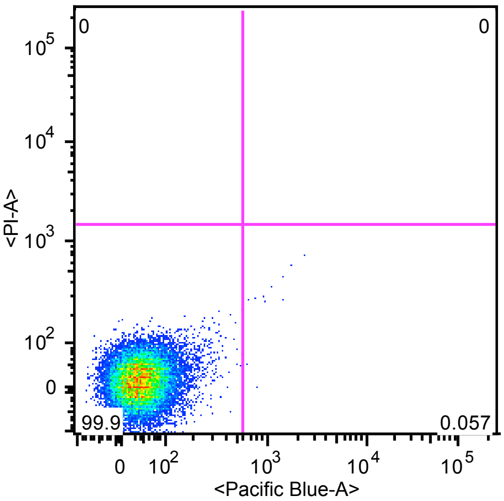

transfected cells
HCT116 wt_GFP_only.fcs
Event Count: 22916

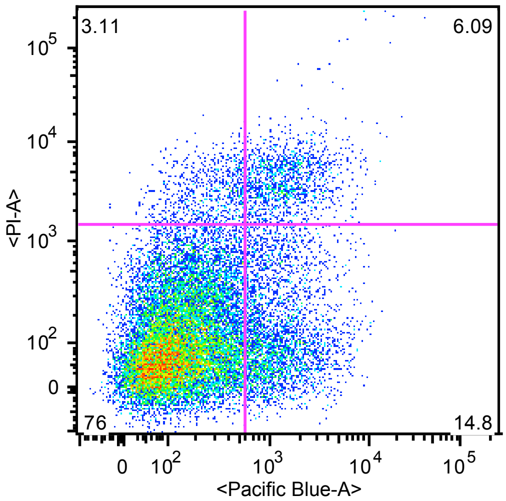

transfected cells
HCT116 wt_GFP Annexin PI.fcs
Event Count: 22606

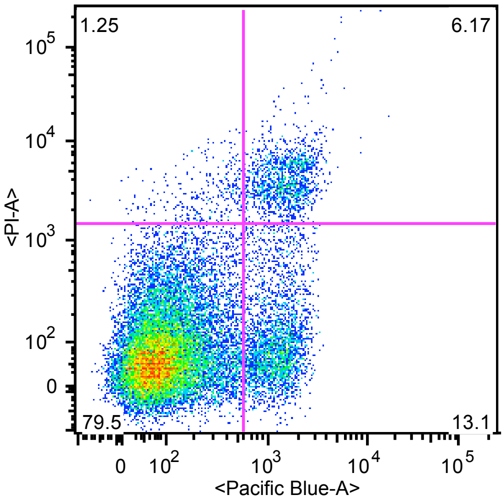

transfected cells
HCT116 wt_Myc.fcs
Event Count: 23130

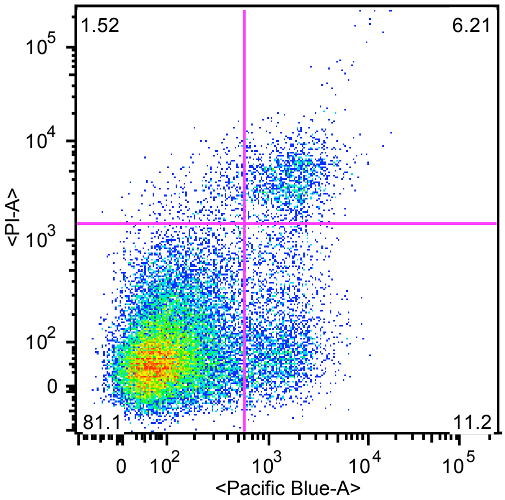

transfected cells
HCT116 wt_shMyc.fcs
Event Count: 23431
